# Activity of Uncleaved Caspase-8 Controls Anti-bacterial Immune Defense and TLR-Induced Cytokine Production Independent of Cell Death

**DOI:** 10.1371/journal.ppat.1005910

**Published:** 2016-10-13

**Authors:** Naomi H. Philip, Alexandra DeLaney, Lance W. Peterson, Melanie Santos-Marrero, Jennifer T. Grier, Yan Sun, Meghan A. Wynosky-Dolfi, Erin E. Zwack, Baofeng Hu, Tayla M. Olsen, Anthony Rongvaux, Scott D. Pope, Carolina B. López, Andrew Oberst, Daniel P. Beiting, Jorge Henao-Mejia, Igor E. Brodsky

**Affiliations:** 1 University of Pennsylvania School of Veterinary Medicine, Department of Pathobiology, Philadelphia, Pennsylvania, United States of America; 2 University of Pennsylvania Perelman School of Medicine, Institute for Immunology, Philadelphia, Pennsylvania, United States of America; 3 University of Washington, Department of Immunology, Seattle, Washington, United States of America; 4 Fred Hutchinson Cancer Research Center, Clinical Research Division and Program in Immunology, Seattle, Washington, United States of America; 5 Yale University School of Medicine, Department of Immunobiology, New Haven, Connecticut, United States of America; 6 Department of Pathology and Laboratory Medicine, Perelman School of Medicine at the University of Pennsylvania and Children’s Hospital of Philadelphia, Philadelphia, Pennsylvania, United States of America; Emory University School of Medicine, UNITED STATES

## Abstract

Caspases regulate cell death programs in response to environmental stresses, including infection and inflammation, and are therefore critical for the proper operation of the mammalian immune system. Caspase-8 is necessary for optimal production of inflammatory cytokines and host defense against infection by multiple pathogens including *Yersinia*, but whether this is due to death of infected cells or an intrinsic role of caspase-8 in TLR-induced gene expression is unknown. Caspase-8 activation at death signaling complexes results in its autoprocessing and subsequent cleavage and activation of its downstream apoptotic targets. Whether caspase-8 activity is also important for inflammatory gene expression during bacterial infection has not been investigated. Here, we report that caspase-8 plays an essential cell-intrinsic role in innate inflammatory cytokine production *in vivo* during *Yersinia* infection. Unexpectedly, we found that caspase-8 enzymatic activity regulates gene expression in response to bacterial infection as well as TLR signaling independently of apoptosis. Using newly-generated mice in which caspase-8 autoprocessing is ablated (*Casp8* ^*DA/DA*^), we now demonstrate that caspase-8 enzymatic activity, but not autoprocessing, mediates induction of inflammatory cytokines by bacterial infection and a wide variety of TLR stimuli. Because unprocessed caspase-8 functions in an enzymatic complex with its homolog cFLIP, our findings implicate the caspase-8/cFLIP heterodimer in control of inflammatory cytokines during microbial infection, and provide new insight into regulation of antibacterial immune defense.

## Author Summary

TLR signaling induces expression of key inflammatory cytokines and pro-survival factors that facilitate control of microbial infection. TLR signaling can also engage cell death pathways through activation of enzymes known as caspases. Caspase-8 activates apoptosis in response to infection by pathogens that interfere with NF-κB signaling, including *Yersinia*, but has also recently been linked to control of inflammatory gene expression. Pathogenic *Yersinia* can cause severe disease ranging from gastroenteritis to plague. While caspase-8 mediates cell death in response to *Yersinia* infection as well as other signals, its precise role in gene expression and host defense during *in vivo* infection is unknown. Here, we show that caspase-8 activity promotes cell-intrinsic cytokine expression, independent of its role in cell death in response to *Yersinia* infection. Our studies further demonstrate that caspase-8 enzymatic activity plays a previously undescribed role in ensuring optimal TLR-induced gene expression by innate cells during bacterial infection. This work sheds new light on mechanisms that regulate essential innate anti-bacterial immune defense.

## Introduction

Pattern recognition receptors such as Toll-like receptors (TLRs) sense conserved microbial structures including lipopolysaccharide (LPS) or peptidoglycans [1]. Bacterial infection triggers MyD88- and TRIF-dependent MAPK and NF-κB signaling, which induces the expression of cell survival and inflammatory programs that are critical for host defense [2]. Activation of TLRs in the presence of pharmacological or bacterial inhibitors of NF-κB results in cell death that is mediated by the cysteine protease caspase-8 [3–5]. This is due to recruitment of caspase-8 to a TRIF/RIPK1/FADD-containing complex via specific homotypic protein-protein interaction motifs [6]. RIPK1 interacts with TRIF by means of RIP homology interaction motifs (RHIM) and can bind FADD through shared death domains (DD), which in turn engages caspase-8 via death effector domains (DED) [7, 8]. Upon recruitment to this complex, caspase-8 undergoes dimerization and autoprocessing, which stabilizes the active enzyme, and initiates the proteolytic cascade that ultimately results in apoptotic disassembly of the cell [9].

Spontaneous mutations in human caspase-8 that render it catalytically inactive are linked with primary immunodeficiency and recurrent sinopulmonary and mucocutaneous infections [10, 11]. Similarly, individuals with mutations in the adaptor FADD suffer from recurrent infections and liver pathology, suggesting a role for caspase-8 and FADD in antimicrobial responses [12]. Initial studies observed that T, B and NK cells from patients with caspase-8 deficiency displayed defects in activation following stimulation through their cell-type specific functional receptors [10, 13]. Interestingly, reconstitution of a caspase-8-deficient Jurkat T cell line implicated the enzymatic activity of uncleaved caspase-8 in activation of T cells via TCR [13]. However, subsequent work revealed that caspase-8 is critical to protect T cells from programmed necrosis in the setting of TCR stimulation, and that rescuing this survival defect restored the ability of T cells to respond to viral infection [14, 15]. These studies suggested the possibility that the effect of caspase-8 on activation could relate to its control of cell death, rather than activation of transcriptional signaling machinery per se.

The survival function of caspase-8 prevents receptor-interacting serine/threonine protein kinase-3 (RIPK3)-dependent necroptosis, which occurs in the context of developmental and inflammatory cues [15–20]. During homeostasis, RIPK3 is repressed by heterodimers of caspase-8 and its catalytically inactive homologue, cFLIP. However, inhibition of caspase-8 activity or deletion of caspase-8 releases RIPK3-dependent necroptosis [15, 18]. TLR signaling normally prevents caspase-8-, FADD- and RIPK3-dependent cell death pathways both through transcriptional upregulation of pro-survival genes and through post-translational modification of key signaling proteins such as RIPK1 [21], as well as inducing caspase-8-dependent cleavage of the pro-necroptotic molecule CYLD [22]. Cell death and inflammatory gene expression are therefore thought to be mutually exclusive programs. However, recent studies have revealed that caspase-8 nonetheless regulates innate anti-microbial responses [23–29]. These studies have primarily investigated the function of caspase-8 in the context of RIPK3 deficiency, and have not addressed the potential scaffolding and enzymatic activities of caspase-8 in controlling these distinct functions. Moreover, it is unclear whether caspase-8 plays a cell-intrinsic role in controlling gene expression *in vivo* during bacterial infection.

Combined deficiency of caspase-8 or FADD and RIPK3 leads to significant reduction in the secretion of a number of pro-inflammatory mediators as well as loss of inflammasome priming and activation in response to some stimuli [23, 24, 26, 29]. Interestingly, while many of these inflammatory mediators are regulated by the NF-κB signaling pathway, whether caspase-8 regulates proximal NF-κB signaling, and even whether caspase-8 acts as a negative or positive regulator of inflammatory gene expression remains unresolved, due to the coupling of caspase-8 deficiency with induction of programmed necrosis. Thus, these studies take place either under conditions where RIPK3 is ablated [23, 24, 26, 29], or under conditions where programmed necrosis can occur in cells with conditional deletion of caspase-8 [13, 30–32]. How caspase-8 might function to regulate both cell death and inflammatory gene expression, and whether enzymatic activity plays a role in the latter response is currently unknown.

Here we demonstrate that caspase-8 enzymatic activity is necessary for cell-intrinsic control of key inflammatory cytokine gene expression in response to gram-negative bacterial infection as well as multiple TLR agonists. We found that regulation of gene expression by caspase-8 was independent of cell death and caspase-8 apoptotic substrates. Notably, caspase-8 controlled expression of a key subset of TLR-induced genes that regulate inflammation and host defense. To dissect the contribution of caspase-8 activity to cytokine gene expression, we generated CRISPR-based caspase-8 knock-in mice in which the self-cleavage of caspase-8 was abrogated due to a mutation in aspartate 387 to alanine (*Casp8* ^*DA/DA*^). Intriguingly, *Casp8* ^*DA/DA*^ macrophages could not undergo caspase-8-dependent apoptosis, but were functional for caspase-8-dependent control of inflammatory cytokine expression. As *Casp8* ^*DA/DA*^ has no known enzymatic activity in the absence of the cFLIP, our findings implicate a novel function for the caspase-8/cFLIP heterodimer in induction of innate inflammatory gene expression and provide insight into control of antimicrobial host defense.

## Results

### Caspase-8 plays a cell-intrinsic role in inflammatory cytokine production during *Yersinia* infection *in vivo*

**Fig 1:**
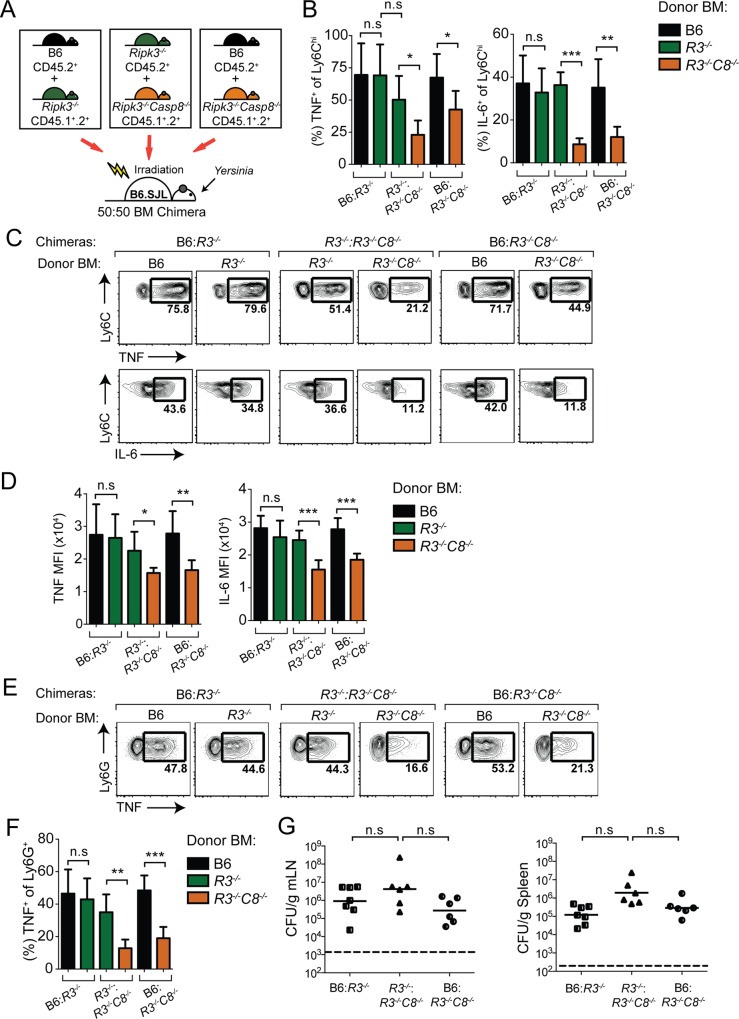
*Ripk3* ^*-/-*^ *Casp8* ^*-/-*^ inflammatory monocytes and neutrophils have a cell-intrinsic defect in IL-6 and TNF production. (A) Schematic of mixed bone marrow chimera experimental set-up. Congenically marked B6 (black), *Ripk3* ^*-/-*^ (green) or *Ripk3* ^*-/-*^ *Casp8* ^*-/-*^ (orange) bone marrow (BM) were injected at a 1:1 ratio into lethally-irradiated recipient B6.SJL mice. 8 weeks after reconstitution, chimeras were orally infected with *Yersinia* (1x10^8^/mouse) and immune responses were assayed at day 5 post-infection. (B) Quantification of percentage of Ly6C^hi^ inflammatory monocytes that express TNF or IL-6 for each genotype of cells in the B6:*Ripk3* ^*-/-*^ (B6:*R3* ^*-/-*^), *Ripk3* ^*-/-*^:*Ripk3* ^*-/-*^ *Casp8* ^*-/-*^ (*R3* ^*-/-*^:*R3* ^*-/-*^ *C8* ^*-/-*^) and B6:*Ripk3* ^*-/-*^ *Casp8* ^*-/-*^ (B6:*R3* ^*-/-*^ *C8* ^*-/-*^) mixed chimeras, as indicated. Color scheme of bars is as in (A) with black bars representing B6, green bars representing *Ripk3* ^*-/-*^, and orange bars representing *Ripk3* ^*-/-*^ *Casp8* ^*-/-*^ cells. (C) Representative flow plots of TNF (top row of plots) and IL-6 (bottom row of plots) production in inflammatory monocytes from mixed chimeras in (B). Flow plots within each set of brackets represent cells analyzed from the same mixed bone marrow recipient mouse; genotypes of the cells analyzed are indicated above each plot *R3* ^*-/-*^–*Ripk3* ^*-/-*^, *R3* ^*-/-*^ *C8* ^*-/-*^—*Ripk3* ^*-/-*^ *Casp8* ^*-/-*^. (D) Quantification of mean fluorescence intensity (MFI) of TNF^+^ and IL-6^+^ inflammatory monocytes from B6:*R3* ^*-/-*^, *R3* ^*-/-*^:*R3* ^*-/-*^ *C8* ^*-/-*^and B6:*R3* ^*-/-*^ *C8* ^*-/-*^ mixed chimeras in (B) and (C). (E) Representative flow plots of TNF production in Ly6G^+^ neutrophils from B6:*R3* ^*-/-*^, *R3* ^*-/-*^:*R3* ^*-/-*^ *C8* ^*-/-*^ and B6:*R3* ^*-/-*^ *C8* ^*-/-*^ mixed chimeras analyzed as described in (C). (F) Quantification of percentage of TNF^+^ neutrophils from (E). (G) Quantification of bacterial burden per gram tissue (CFU/g). Solid bars indicate geometric mean of samples. Dotted lines indicate the limit of detection. Gating strategy is described in detail in Materials and Methods. Data are representative of 4 independently performed *Yersinia* infection experiments using 6–7 mice per experimental group. * *p* < 0.05, ** *p* < 0.01, *** *p* < 0.001 by Student’s unpaired two-tailed *t*-test. See also S1 Fig.

*Ripk3* ^*-/-*^ *Casp8* ^*-/-*^ mice exhibit severely diminished cytokine responses following infection by a number of gram-negative bacterial pathogens, including *Yersinia*, in contrast to *Ripk3* ^*-/-*^ mice, which have no discernible defect [24, 26, 29]. *Yersinia* induces caspase-8-dependent cell death in macrophages, which could promote host defense by release of intracellular alarmins that promote inflammatory cytokine production by bystander cells, or phagocytosis of *Yersinia-*infected apoptotic cells [26, 33]. Alternatively, caspase-8 could have a cell-intrinsic effect on inflammatory gene expression, which has not previously been described *in vivo* during bacterial infection. To distinguish between these possibilities, we generated mixed bone marrow chimeras using transfer of congenically marked WT, *Ripk3* ^*-/-*^, and *Ripk3* ^*-/-*^ *Casp8* ^*-/-*^ donor bone marrow at 1:1 ratios into lethally-irradiated wild-type (B6.SJL) recipients (Fig 1A and S1 Fig). Eight weeks post-reconstitution, mixed chimeric animals were infected with *Yersinia pseudotuberculosis* (Yp), which causes a rapid and lethal bacteremia in animals with hematopoietic caspase-8 deficiency [26, 29]. Strikingly, five days post-infection, we found a significant defect in the percentage of TNF and IL-6 positive *Ripk3* ^*-/-*^ *Casp8* ^*-/-*^ inflammatory monocytes isolated from the mesenteric lymph nodes (mLN) of mixed BM chimeras compared with either wild-type or *Ripk3* ^*-/-*^ cells from the same animal (Fig 1B and 1C and S1B and S1C Fig). Moreover, this defect was equivalent to monocytes from recipients receiving only *Ripk3* ^*-/-*^ *Casp8* ^*-/-*^ BM, indicating that the presence of caspase-8-sufficient cells did not restore cytokine production to caspase-8-deficient cells in the same animal (S1B and S1C Fig). Interestingly, the mean fluorescence intensity (MFI) of *Ripk3* ^*-/-*^ *Casp8* ^*-/-*^ cytokine positive cells was significantly lower than *Ripk3* ^*-/-*^ or B6 cells from the same mouse, indicating a reduced level of cytokine production per cell (Fig 1D). We also observed a significantly lower frequency of *Ripk3* ^*-/-*^ *Casp8* ^*-/-*^ TNF-producing neutrophils compared to either *Ripk3* ^*-/-*^ or wild type neutrophils in the same mouse, indicating that this defect in inflammatory cytokine production *in vivo* extended to other innate cell types (Fig 1E and 1F). The percent chimerism of these cell types was similar across all the genotypes, indicating similar generation and maintenance of *Ripk3* ^*-/-*^ *Casp8* ^*-/-*^ monocytes and neutrophils in a competitive environment (S1D and S1E Fig). Mice reconstituted with *Ripk3* ^*-/-*^ *Casp8* ^*-/-*^ bone marrow cannot control *Yersinia* and harbor much higher bacterial burdens in their lymph nodes and spleen [26, 29]. Importantly, the presence of WT or *Ripk3* ^*-/-*^ cells in the *Ripk3* ^*-/-*^ *Casp8* ^*-/-*^ mixed BM chimeras provided protection from *Yersinia* infection, as the *Ripk3* ^*-/-*^ *Casp8* ^*-/-*^ mixed BM chimeras had similar bacterial burdens compared to mice that contained only wild-type or a mixture of *Ripk3* ^*-/-*^ and wild-type bone marrow (Fig 1G and S1F Fig). These data provide direct evidence that caspase-8 plays a key cell-intrinsic role in inflammatory gene expression during *Yersinia* infection independently of cell death.

### Caspase-8 regulates inflammatory cytokine production by multiple TLRs

**Fig 2:**
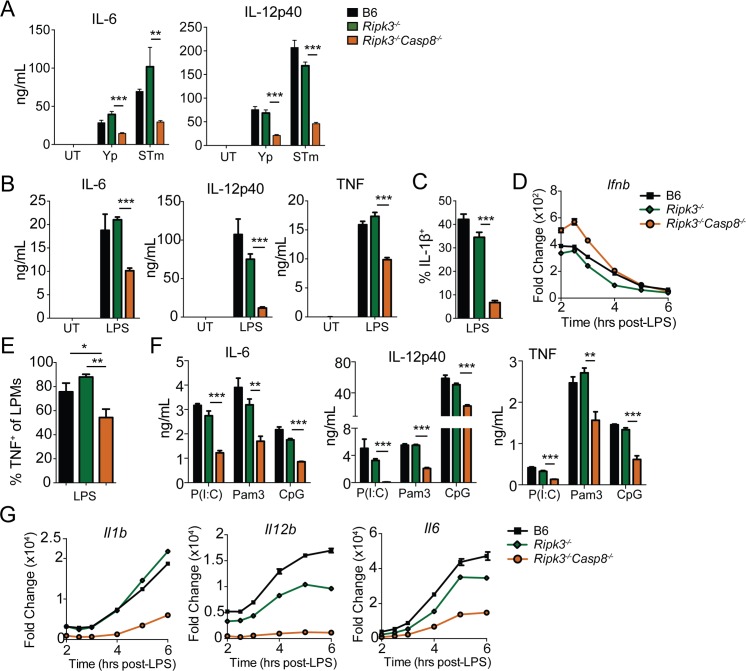
*Ripk3* ^*-/-*^ *Casp8* ^*-/-*^ bone marrow-derived macrophages are defective in both MyD88 and TRIF-dependent cytokine production. (A) Bone marrow-derived macrophages (BMDMs) from B6, *Ripk3* ^*-/-*^ and *Ripk3* ^*-/-*^ *Casp8* ^*-/-*^ were infected with *Yersinia* or *Salmonella* for 24hrs, and IL-6 and IL-12p40 cytokine levels present in supernatants were quantified by ELISA. (B) IL-6, IL-12p40 and TNF production was measured by ELISA from the indicated BMDMs treated with LPS (100 ng/mL) for 6hrs. (C) IL-1β expression was measured by flow cytometry from the indicated BMDMs treated with LPS (100 ng/mL) for 5hrs. (D) BMDMs were treated with LPS (100 ng/mL) and *Ifnb* mRNA was assayed by RT-qPCR at the indicated time points. (E) Resident peritoneal macrophages from indicated mouse genotypes were isolated and stimulated *ex vivo* with LPS (10 ng/mL) for 4 hrs. TNF production by large peritoneal macrophages (LPMs) was measured by flow cytometry. (F) IL-6, IL-12p40 and TNF production was measured by ELISA from BMDMs treated with Poly(I:C) (50 μg/mL) for 24 hrs, CpG (1 μg/mL) or Pam3CSK4 (1 μg/mL) for 6hrs.(G) BMDMs were treated with LPS (100 ng/mL) and *Il1b*, *Il12b* and *Il6* mRNA was assayed by RT-qPCR at the indicated time points. * *p* < 0.05, ** *p* < 0.01, *** *p* < 0.001 by Student's unpaired two-tailed *t*-test. Representative of minimum of three independently performed experiments with triplicate samples for each condition. See also S2 Fig.

To further define this response, we investigated the potential contribution of caspase-8 to cytokine production in response to bacterial infection and individual pathogen-associated molecular patterns (PAMPS) in bone marrow-derived macrophages (BMDMs). *Ripk3* ^*-/-*^ *Casp8* ^*-/-*^ BMDMs showed dramatically reduced IL-6 and IL-12p40 production compared with B6 or *Ripk3* ^*-/-*^ cells in response to *Yersinia* and *Salmonella* infection (Fig 2A). Consistent with this as well as previous observations [23, 24, 26, 29], *Ripk3* ^*-/-*^ *Casp8* ^*-/-*^ BMDMs produced lower levels of secreted IL-6, IL-12p40, and TNF, as well as intracellular pro-IL-1β in response to LPS treatment (Fig 2B and 2C). Importantly, *Ripk3* ^*-/-*^ *Casp8* ^*-/-*^ BMDMs did not have a global defect in LPS-induced responses, as *Ifnb* transcript levels were not decreased, suggesting a more selective effect of caspase-8 deficiency (Fig 2D). Peritoneal macrophages isolated from *Ripk3* ^*-/-*^ *Casp8* ^*-/-*^ mice and stimulated *ex vivo* with LPS also showed a significant defect in TNF production relative to B6 and *Ripk3* ^*-/-*^ peritoneal macrophages (Fig 2E), further supporting a role for caspase-8 in cytokine production by innate cells following TLR stimulation.

Caspase-8 plays a role in control of cytokine responses downstream of TLR4 and TLR3, both of which are coupled to TRIF [23]. Whether MyD88-dependent TLRs also engage caspase-8 for maximal cytokine production is not known. Surprisingly, RIPK3/caspase-8-deficient BMDMs exhibited reduced production of IL-6, IL-12p40, and TNF in response to the MyD88-dependent TLR2 and TLR9 ligands Pam3CSK4 and CpG, as well as the TRIF-dependent TLR3 agonist Poly(I:C) (Fig 2F). These data demonstrate that caspase-8 mediates gene expression downstream of both TRIF- and MyD88-dependent pathways.

These cytokines depend on NF-κB and AP-1 transcription factor family members, and previous studies have suggested that caspase-8 regulates the activation of NF-κB [13, 29, 31]. At what step and how caspase-8 might regulate NF-κB is nevertheless unclear. TLR signaling induces assembly of MyD88 into a complex with proteins of the IRAK family called the Myddosome [34, 35], which initiates the signaling events that lead to the activation of NF-κB transcription factors to mediate NF-κB-dependent cytokine and chemokine gene expression [34, 35]. However, we did not detect caspase-8 in the Myddosome, and caspase-8 did not affect IRAK2 localization to the Myddosome in response to LPS-treatment (S2A and S2B Fig), suggesting that caspase-8 is not involved in TLR-proximal signaling events. Initial studies had observed a role for caspase-8 in regulating the NF-κB pathway in lymphocytes [13, 31], and Weng *et al*. reported moderate effects on IκBα degradation in *Ripk3* ^*-/-*^ *Casp8* ^*-/-*^ macrophages that could potentially account for a defect in cytokine production. Nevertheless, consistent with Allam *et al*. [23], degradation and resynthesis of IκBα in our hands was similar among LPS-treated B6, *Ripk3* ^*-/-*^, and *Ripk3* ^*-/-*^ *Casp8* ^*-/-*^ BMDMs at these timepoints (S2C Fig). Moreover, other components of TLR signaling pathways including AKT and MAPK activation were not affected by caspase-8, as we observed similar levels of AKT or p38 in LPS-treated *Ripk3* ^*-/-*^ *Casp8* ^*-/-*^ BMDMs compared to B6 and *Ripk3* ^*-/-*^ BMDMs (S2D and S2E Fig). Finally, recruitment of the NF-κB family member p65 to caspase-8-inducible gene promoters was not affected in *Ripk3* ^*-/-*^ *Casp8* ^*-/-*^ BMDMs (S2F Fig). Together, these data suggest that the role of caspase-8 in gene expression likely does not occur via receptor-proximal effects on classical NF-κB or MAPK signaling.

Caspase-8 could potentially regulate inflammatory cytokine production by inducing mRNA transcription, or by promoting mRNA stability. Indeed, LPS-stimulated *Ripk3* ^*-/-*^ *Casp8* ^*-/-*^ BMDMs had significantly lower levels of *Il1b*, *Il12b* and *Il6* mRNA transcript compared to *Ripk3* ^*-/-*^ or wild type BMDMs (Fig 2G). Importantly however, *Ripk3* ^*-/-*^ *Casp8* ^*-/-*^ BMDMs exhibited equal levels of mRNA stability following treatment with the transcription synthesis inhibitor actinomycin D (ActD), indicating that the reduced transcript levels in *Ripk3* ^*-/-*^ *Casp8* ^*-/-*^ cells were likely due to decreased transcriptional induction (S3 Fig).

### Caspase-8-regulated genes are enriched for cytokine signaling pathways

**Fig 3:**
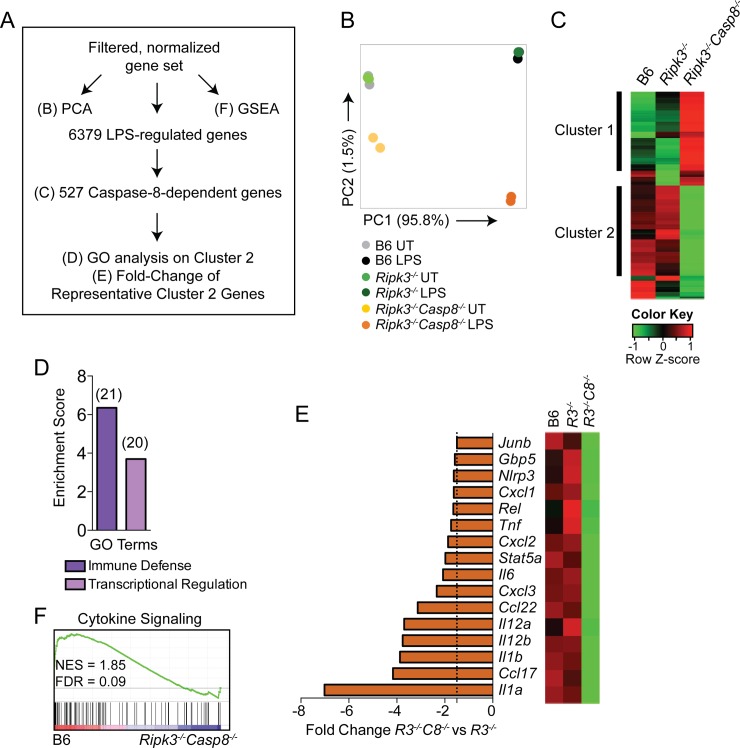
Caspase-8 regulates a functionally important subset of LPS-induced genes. RNA was extracted from B6, *Ripk3* ^*-/-*^ and *Ripk3* ^*-/-*^ *Casp8* ^*-/-*^ BMDMs following 6 hours of LPS treatment (100 ng/mL) and RNA-seq was performed. (A) Schematic of analysis of the data obtained from RNA-seq indicating figure panel where the results of each type of analysis is shown. (B) Principal Component Analysis (PCA) of filtered, normalized gene set displaying PC1 (95.8% of variance) against PC2 (1.5% of variance). (C) Hierarchical clustering by Pearson correlation of differentially expressed LPS-responsive caspase-8-dependent genes. Columns represent genotype and rows represent individual genes. Colored to indicate expression levels based on Z-scores. (D) GO enrichment performed in DAVID showing Biological Process terms enriched in cluster 2 from (C). Number of genes in each group are denoted above bars. Genes in cluster 1 did not show significant enrichment for any Biological Process terms. (E) Differential expression of select genes from cluster 2 and fold change (LPS-treated *Ripk3* ^*-/-*^ *Casp8* ^*-/-*^ vs LPS-treated *Ripk3* ^*-/-*^ BMDMs). Dotted line represents 1.5-fold cutoff. (F) GSEA showing enrichment in cytokine signaling from the KEGG MSigDb canonical pathways collection 2 comparing B6 and *Ripk3* ^*-/-*^ *Casp8* ^*-/-*^ LPS-treated BMDMs. GO, gene ontology; DAVID, database for annotation visualization and integrated discovery; GSEA, gene set enrichment analysis; KEGG, Kyoto Encyclopedia of Genes and Genome; NES, normalized enrichment score; FDR, false discovery rate. *R3C8* = *Ripk3* ^*-/-*^ *Casp8* ^*-/-*^. See also S3 and S4 Figs.

Only a limited set of TLR-induced responses have been described to be regulated by caspase-8 [23–26, 28, 29]. To address the extent to which caspase-8 regulates TLR-induced gene expression, we transcriptionally profiled B6, *Ripk3* ^*-/-*^, and *Ripk3* ^*-/-*^ *Casp8* ^*-/-*^ BMDMs following LPS treatment. Based on our observations that maximal differences between *Ripk3* ^*-/-*^ *Casp8* ^*-/-*^ and B6 or *Ripk3* ^*-/-*^ BMDMs occurred at 6 hours post-infection, we performed RNA-seq analysis on these three genotypes of macrophages 6 hours after LPS stimulation. This timepoint also allowed for detection of both primary and secondary response genes [36]. To define the contribution of caspase-8 to the TLR4-induced transcriptional program, we analyzed the LPS-induced genes by Principle Component Analysis (PCA), Gene Set Enrichment Analysis (GSEA), hierarchical clustering, and Gene Ontology (GO) analysis (Fig 3A). The PCA revealed that LPS treatment accounted for almost 96% of the variance among the samples (PC1), demonstrating that neither RIPK3 deficiency alone, nor combined deficiency of caspase-8 and RIPK3 globally affected LPS-induced gene expression (Fig 3B). However, RIPK3/caspase-8-deficiency contributed to the second highest variance (PC2), implying a role for caspase-8 in LPS-induced gene expression (Fig 3B). To determine the precise contribution of caspase-8 to the LPS response, we identified LPS-regulated genes that were altered in either *Ripk3* ^*-/-*^ *Casp8* ^*-/-*^ or *Ripk3* ^*-/-*^ BMDMs. Interestingly, 527 (8.3%) of the 6379 genes were RIPK3/caspase-8-dependent, whereas only 62 of 6379 (less than 1%) were RIPK3-dependent (S2 Text and S1 Table). Importantly, 479 (91%) of the 527 genes affected in *Ripk3* ^*-/-*^ *Casp8* ^*-/-*^ BMDMs were unaffected by RIPK3 deficiency alone, implying a specific role for caspase-8 in induction of these genes. Hierarchical clustering of the caspase-8-dependent genes by Pearson correlation revealed two clusters of coordinately regulated genes (Fig 3C). Genes in cluster 1 were more highly expressed in response to LPS in *Ripk3* ^*-/-*^ *Casp8* ^*-/-*^ BMDMs, whereas cluster 2 was composed of genes that were not as strongly induced by LPS stimulation in *Ripk3* ^*-/-*^ *Casp8* ^*-/-*^ BMDMs compared to B6 or *Ripk3* ^*-/-*^ BMDMs. Functional enrichment of cluster 2 genes using Gene Ontology (GO) analysis revealed genes associated with immune defense and transcriptional regulation (Fig 3D). Genes belonging to the category of transcriptional regulation included *JunB*, *Rel* and *Stat5a* (Fig 3E), while the category of immune defense included *Il1a*, *Il1b*, *Ccl17*, *Il12b*, *and Tnf* (Fig 3E). Although this subset of genes was still upregulated by LPS stimulation in *Ripk3* ^*-/-*^ *Casp8* ^*-/-*^ BMDMs, the degree of induction was significantly reduced relative to control cells (S4A Fig). Intriguingly, *Il1a*, *Il1b*, *Ccl17* and *Il12b* were among the top 20 most differentially expressed genes in the absence of caspase-8. Furthermore, Gene Set Enrichment Analysis (GSEA), which provides an unbiased way to identify coordinated changes in gene expression, demonstrated that genes involved in cytokine and chemokine signaling were significantly enriched in wild type BMDMs compared to caspase-8-deficient cells (Fig 3F). These findings demonstrate that caspase-8 deficiency is associated with altered transcriptional responses of LPS-induced genes that play a particularly important role in inflammatory responses to infection.

Caspase-8 has been reported to localize to the nucleus of B cells, raising the possibility that nuclear caspase-8 might play a role in gene expression [37]. However, we did not observe caspase-8 in the nucleus, despite seeing robust nuclear localization of the AP-1 family member JunB, suggesting that caspase-8 does not play a direct role in transcriptional activation of these target genes (S4B Fig). Furthermore, both *Ripk3* ^*-/-*^ and *Ripk3* ^*-/-*^ *Casp8* ^*-/-*^ BMDMs phagocytosed and cleared *Salmonella* and *Yersinia* equivalently to wild-type cells, indicating that direct microbicidal mechanisms remain largely intact in *Ripk3* ^*-/-*^ *Casp8* ^*-/-*^ macrophages (S4C Fig). Together, these data indicate that the loss of caspase-8 is associated with altered expression of a significant number of LPS-induced genes that play a critical role in inflammatory responses, potentially providing an explanation for the profound susceptibility of caspase-8-deficient animals to bacterial pathogens.

TLRs, TNFR, and the IL-1R receptor family engage both shared and specific signaling modules to induce gene expression programs [38, 39]. Notably, IL-1R and TLRs utilize the shared signaling adaptor MyD88. Nevertheless, TNF and IL-1β stimulation does not induce the same genes in macrophages that are induced by TLRs, indicating important distinctions exist in the way that cells respond to these extracellular cues [39–41]. Indeed, whereas TLR signaling induces a large number of genes including inflammatory cytokines as well as chemokines in macrophages, stimulation of IL-1R and TNFR alone primarily induces expression of chemokines but not cytokines [38–40]. In order to test the potential role of caspase-8 in induction of IL-1R or TNFR-dependent genes, we therefore examined genes that were commonly induced in BMDMs by TLR4, IL-1R, or TNFR stimulation. Interestingly, despite the role of caspase-8 in regulating cell death and survival decisions in macrophages in the context of TNFR as well as TLR signaling [42], caspase-8 deletion did not affect the expression of *Cxcl2* or *Ccl22* in response to TNF, even though caspase-8 was necessary for optimal expression of these genes downstream of TLR engagement (S4D and S4E Fig). Similarly, *Cxcl1*, whose induction requires caspase-8 following TLR stimulation, was induced to a similar degree and with similar kinetics in response to IL-1R engagement in caspase-8-deficient BMDMs (S4F Fig).

Caspase-8 was previously shown to negatively regulate type I IFN in response to Sendai Virus (SeV) in human fibroblasts, in part through cleaving RIPK1 and shutting off RIPK1 signaling [27]. However, caspase-8 was dispensable for SeV-induced expression of IL-6 and IL-12p40 in BMDMs (S5A Fig). *Ripk3* ^*-/-*^ *Casp8* ^*-/-*^ mice also had equivalent levels of cytokine responses in the lung, similar viral burdens, and similar kinetics of weight loss over the course of the infection (S5B–S5D Fig). Thus, while caspase-8 is important for maximal inflammatory gene expression in response to bacterial infection, it is dispensable for innate responses against and clearance of SeV.

### Caspase-8 catalytic activity, but not autoprocessing, is required for optimal TLR-induced cytokine production

**Fig 4:**
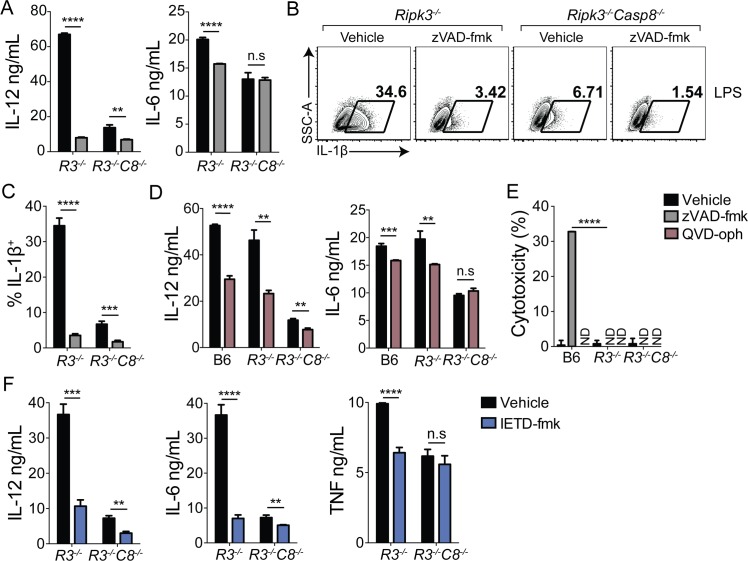
Caspase-8 catalytic activity is required for maximal TLR-induced cytokine production. (A-F) Indicated BMDMs were pretreated with the pan-caspase inhibitor zVAD-fmk (A-C), QVD-oph (D) or IETD-fmk (F) for 1hr prior to 6 hr stimulation with LPS (100 ng/mL). TNF (F), IL12p40 (A, E, F) and IL-6 (A, E, F) were measured by ELISA, IL-1β was measured by flow cytometry (B, C) and cytotoxicity was measured by lactate dehydrogenase release (LDH) (E). All inhibitors were used at 100 μM. ** *p* < 0.01, *** *p* < 0.001, **** *p* < 0.0001. Student’s unpaired two-tailed *t*-test. Representative of 4 or more independent experiments. See also S6 Fig.

Caspase-8 can be recruited to multiple protein complexes that carry out diverse functions and are assembled in response to specific extracellular cues. Caspase-8-dependent activation of caspase-1 during NLRP3 inflammasome activation does not depend on caspase activity, suggesting that in this context, caspase-8 plays a scaffolding role, potentially by recruiting caspase-1 to undergo auto-processing [43]. However, whether caspase-8 enzymatic activity is important for induction of inflammatory gene expression is currently unclear [13, 14]. Caspase-8 homodimerization results in activation of apoptosis, whereas heterodimerization of caspase-8 with its catalytically inactive homolog cFLIP prevents both apoptosis and regulated necrosis [18, 44]. Conditions that induce extrinsic apoptosis induce assembly of caspase-8 homodimers or oligomers, wherein caspase-8 undergoes autoprocessing [45, 46]. While homodimerization is sufficient to activate the enzyme, subsequent autoprocessing stabilizes the cleaved dimer, and is required for caspase-8 to cleave its downstream apoptotic targets, Bid, caspase-3 and -7 [9]. In contrast, the caspase-8/cFLIP heterodimer requires catalytic activity but not autoprocessing to prevent necrosis [18]. Blocking caspase activity in the context of TNF or TLR stimulation therefore leads to programmed necrosis that depends on RIPK3 [47]. As RIPK3-deficient cells do not undergo cell death in response to blockade of caspase activity, we treated RIPK3-deficient cells with the pan-caspase inhibitor zVAD-fmk and the caspase-8-specific inhibitor IETD-fmk to address whether caspase-8 scaffolding or enzymatic activities were responsible for caspase-8-dependent gene expression. Intriguingly, zVAD-fmk significantly reduced production of IL-12p40, IL-6 and pro-IL-1β in LPS-treated *Ripk3* ^*-/-*^ BMDMs (Fig 4A–4C). Notably, *Ripk3* ^*-/-*^ *Casp8* ^*-/-*^ BMDMs exhibited significantly blunted responses to LPS, which were not substantially altered by inhibitor treatment. QVD-oph is another widely used pan-caspase inhibitor that does not display the same cytotoxicity as zVAD-fmk [48]. Critically, QVD-oph also significantly reduced IL-12p40 and IL-6 production following LPS treatment in both B6 and *Ripk3* ^*-/-*^ BMDMs (Fig 4D). QVD-oph did not induce cytotoxicity in either LPS-treated or untreated B6 or *Ripk3* ^*-/-*^ BMDMs, and as expected, *Ripk3* ^*-/-*^ cells did not undergo cell death in response to zVAD-fmk (Fig 4E). Importantly, in addition to broad spectrum caspase inhibitors, specific inhibition of caspase-8 activity with IETD-fmk in *Ripk3* ^*-/-*^ BMDMs also significantly blunted IL-12, IL-6 and TNF production (Fig 4F). These findings demonstrate that caspase activity contributes to LPS-induced gene expression independently of cell death. Moreover, BMDMs lacking both caspase-3 and -7 or treated with the caspase-3/7 inhibitor DEVD-fmk, were competent to induce inflammatory cytokine expression following LPS treatment, demonstrating that caspase-8 activity regulates TLR-induced gene expression independent of its downstream apoptotic caspases (S6 Fig).

### Caspase-8 is necessary for optimal cytokine expression independently of RIPK3 deficiency

**Fig 5:**
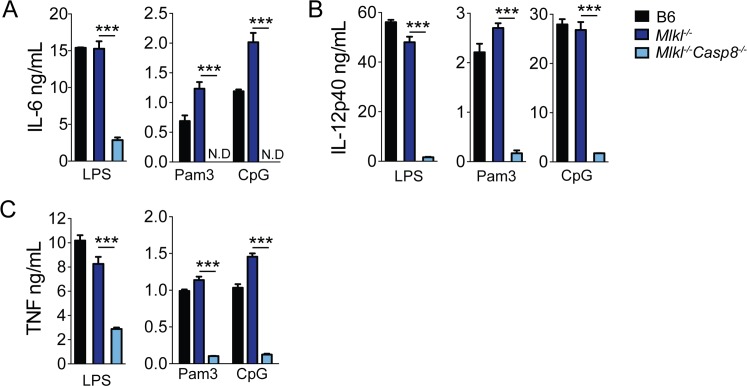
Caspase-8 regulates optimal cytokine expression independently of RIPK3 deficiency. *B6*, *Mlkl* ^*-/-*^ and *Mlkl* ^*-/-*^ *Casp8* ^*-/-*^ BMDMs were treated with LPS (100 ng/mL), Pam3CSK4 (1 μg/mL) or CpG (1 μg/mL) for 6hrs. IL-6 (A), IL-12p40 (B) and TNF (C) release was assayed by ELISA. *** *p* < 0.001 by Student's unpaired two-tailed *t*-test. Representative of two independent experiments.

While caspase-8 inhibits RIPK3-regulated necrosis via a caspase-8/cFLIP complex, mutation of RIPK3 or inhibition of RIPK3 kinase activity can also promote caspase-8-dependent apoptosis [8, 49–51]. Given that under some circumstances, RIPK3 can potentiate some functions of caspase-8, the impact of caspase-8 deficiency on gene expression could be the result of a combined loss of both RIPK3 and caspase-8. However, due to the RIPK3-induced embryonic lethality of caspase-8-deficient animals, the effect of single deficiency in caspase-8 has been difficult to isolate. Conditional deletion of caspase-8 *in vitro* also results in significant toxicity due to induction of RIPK3-mediated necrosis even in cultured cells [52]. To address the possibility that a dual contribution of RIPK3 and caspase-8 was responsible for the deficiency in TLR-induced gene expression in *Ripk3* ^*-/-*^ *Casp8* ^*-/-*^ BMDMs, we sought to delete caspase-8 while maintaining RIPK3 expression. Critically, deletion of caspase-8 in the presence of RIPK3 is possible in the absence of Mixed Lineage Kinase Like (MLKL), which is an essential effector of RIPK3-dependent necrosis [53–55]. Intriguingly, *Mlkl* ^*-/-*^ *Casp8* ^*-/-*^ BMDMs also produced significantly lower levels of IL-6, IL-12p40 and TNF in response to LPS, Pam3CSK4 and CpG, relative to *Mlkl* ^*-/-*^ BMDMs (Fig 5A–5C). These data demonstrate that caspase-8 controls cytokine expression independently of RIPK3.

### Caspase-8 autoprocessing regulates macrophage apoptosis but is not required for TLR-induced cytokine expression

**Fig 6:**
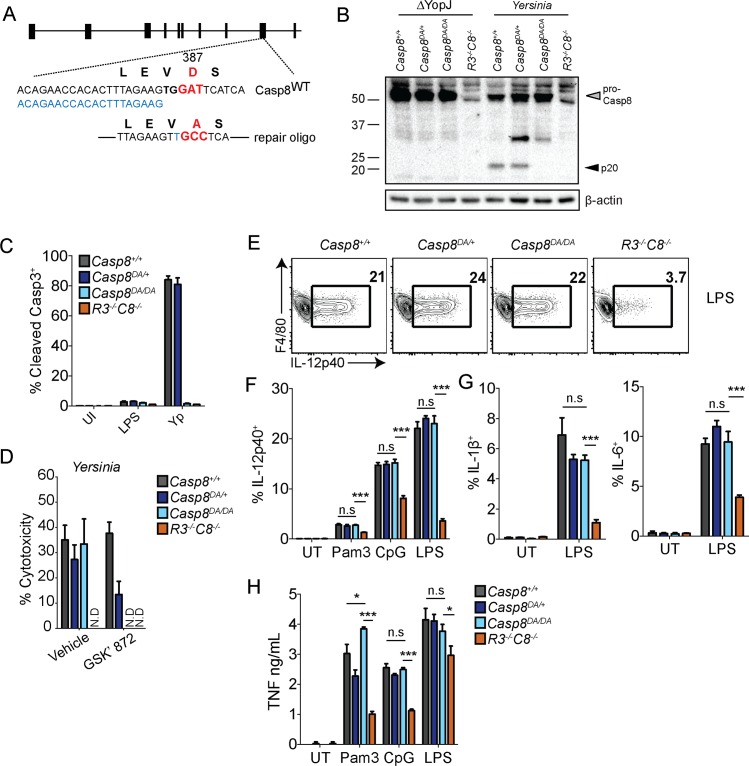
Caspase-8 self-cleavage is necessary for apoptosis but not cytokine responses. (A) Schematic of strategy used to generate *Casp8* ^*DA/DA*^ mice using CRISPR/Cas9. GuideRNA is in blue. (B) *Casp8* ^*+/+*^, *Casp8* ^*DA/+*^, *Casp8* ^*DA/DA*^ and *Ripk3* ^*-/-*^ *Casp8* ^*-/-*^ BMDMs were infected with YopJ-deficient (ΔYopJ) and wild type *Yersinia* and caspase-8 processing was measured by western analysis. (C) BMDMs were pretreated with GSK’ 872 or vehicle control 1 hr prior to infection with *Yersinia*. Cytotoxicity was measured by LDH release 4 hrs post-infection. (D) Cleaved caspase-3 was measured by flow cytometry in BMDMs 2 hrs post-indicated treatments. (E-G) *Casp8* ^*+/+*^, *Casp8* ^*DA/+*^, *Casp8* ^*DA/DA*^ and *Ripk3* ^*-/-*^ *Casp8* ^*-/-*^ BMDMs were treated with PAMPs and cytokine production was measured after 6 hrs by flow cytometry. Representative flow plots of IL-12p40 production in response to LPS (100 ng/mL) (E), quantification of percentage of IL-12p40^+^ in response to LPS (100 ng/mL), Pam3CSK4 (1 μg/mL) or CpG (1 μg/mL) (F), IL-1β^+^ and IL-6^+^ in response to LPS (100 ng/mL) (G) (H) BMDMs were treated with LPS (100 ng/mL), Pam3CSK4 (1 μg/mL) or CpG (1 μg/mL) for 6 hrs and TNF production was measured by ELISA. * *p* < 0.05, ** *p* < 0.01, *** *p* < 0.001, **** *p* < 0.0001. Student’s unpaired two tailed *t*-test. Representative of 3–5 independent experiments.

Caspase-8 activity could potentially regulate TLR-induced gene expression either in the context of a cleaved caspase-8 homodimer, or as a caspase-8/cFLIP heterodimer. To distinguish between these possibilities and to gain further insight into how caspase-8 regulates TLR-induced gene expression, we generated a caspase-8 mutant knock-in mouse, in which aspartate 387 is replaced with an alanine residue (*Casp8* ^*DA/DA*^) (Fig 6A). Although caspase-8 deficiency is embryonically lethal, mice expressing non-cleavable caspase-8 are viable, because the enzymatic activity of caspase-8 in the context of a caspase-8/cFLIP heterodimer inhibits programmed necrosis [18, 56, 57]. Interestingly, this heterodimer can also process caspase-8 substrates, and the substrate preference of caspase-8/cFLIP differs from that of the caspase-8 homodimer [58, 59]. Therefore, the *Casp8* ^*DA/DA*^ mouse provides a means to distinguish the roles of the caspase-8 homodimer and caspase-8/cFLIP heterodimer in gene expression. Notably, *Casp8* ^*DA/DA*^ BMDMs were unable to process caspase-8 in response to *Yersinia*, which induces caspase-8 cleavage and cell death in wild type or *Casp8* ^*DA/+*^ macrophages that depends on the *Yersinia* effector protein YopJ (Fig 6B–6D) [60]. Importantly, *Yersinia-*induced caspase-3 cleavage, which is caspase-8-dependent [26] is specifically abrogated in *Casp8* ^*DA/DA*^ BMDMs (Fig 6C). Surprisingly, despite the absence of caspase-3 cleavage, *Casp8* ^*DA/DA*^ macrophages exhibited equivalent levels of cell death in response to *Yersinia* infection (Fig 6D). Critically, *Casp8* ^*DA/DA*^ BMDMs but not *Casp8* ^*+/+*^ BMDMs were protected from *Yersinia-*induced cell death upon treatment with the RIPK3 inhibitor, GSK’ 872 (Fig 6D). Together, these data demonstrate that caspase-8 cleavage is necessary for *Yersinia-*induced apoptosis in macrophages, and in the absence of this cleavage, *Yersinia-*infected *Casp8* ^*DA/DA*^ BMDMs undergo RIPK3-dependent necrosis. These data imply that caspase-8 D387A does not activate apoptosis, but likely forms a heterodimer with cFLIP in macrophages.

Interestingly, *Casp8* ^*DA/DA*^ and *Casp8* ^*DA/+*^ BMDMs responded equally well to stimulation with LPS, Pam3CSK4 or CpG compared to littermate control WT BMDMs, as we observed similar frequencies of IL-12p40^+^, IL-1β^+^ and IL-6^+^ cells among *Casp8* ^*+/+*^, *Casp8* ^*DA/+*^ and *Casp8* ^*DA/DA*^ BMDMs, and *Casp8* ^*DA/DA*^ BMDMs produced WT levels of TNF in response to LPS, Pam3CSK4 or CpG (Fig 6E–6H). Altogether, our findings indicate that caspase-8 enzymatic, but not its auto-processing, activity plays an important role in optimal production of TLR-dependent inflammatory cytokines.

### cFLIP is necessary for optimal TLR-induced cytokine production

**Fig 7:**
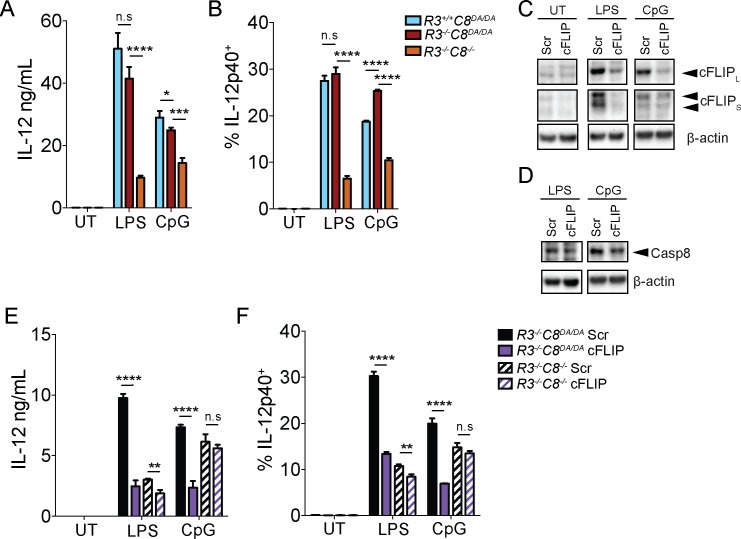
cFLIP is necessary for optimal TLR-induced cytokine production. (A, B) *Ripk3* ^*+/+*^ *Casp8* ^*DA/DA*^, *Ripk3* ^*-/-*^ *Casp8* ^*DA/DA*^, and *Ripk3* ^*-/-*^ *Casp8* ^*-/-*^ BMDMs were treated with LPS (100 ng/mL) or CpG (1 μg/mL), as indicated, and supernatants were analyzed by ELISA for IL-12p40 (A) and frequency of IL-12p40-expressing cells was analyzed by intracellular cytokine staining and flow cytometry (B). (C, D) Primary *Ripk3* ^*-/-*^ *Casp8* ^*DA/DA*^ BMDMs were treated with a pool of cFLIP-specific siRNA or a pool of control scrambled (scr) siRNA (25 nM, Dharmacon) and treated with LPS (100 ng/mL) or CpG (1 μg/mL) or left untreated, as indicated. Whole cell lysates were probed for cFLIP, caspase-8 and β-actin. (E, F) Primary *Ripk3* ^*-/-*^ *Casp8* ^*DA/DA*^ BMDMs were treated as in C, D with a pool of cFLIP-specific siRNA or a pool of scr siRNA and stimulated with LPS (100 ng/mL) or CpG (1 μg/mL), as indicated. IL-12p40 expression in scr and cFLIP siRNA-treated cells was quantified in supernatants by ELISA (E) and intracellularly by intracellular cytokine staining and flow cytometry (F). * *p* < 0.05, ** *p* < 0.01, *** *p* < 0.001, **** *p* < 0.0001. Student’s unpaired two tailed *t*-test. Representative of 3–5 independent experiments.

Caspase-8 autoprocessing is required to stabilize the active enzyme for cleavage of its apoptotic targets [9]. However, uncleaved caspase-8 interacts with cFLIP to prevent programmed necrosis, and the enzymatic activity of the caspase-8/cFLIP heterodimer is required for this function [18]. Whether uncleaved caspase-8 functions together with cFLIP or in a previously undescribed homodimeric complex to mediate inflammatory gene expression has not been determined. Importantly, cFLIP is required to limit both caspase-8-mediated apoptosis and RIPK3-mediated necrosis, as combined deficiency of cFLIP and RIPK3 results in caspase-8-dependent embryonic lethality [56]. To test the potential role of cFLIP in promoting caspase-8-dependent gene expression, we knocked-down cFLIP in *Ripk3* ^*-/-*^ *Casp8* ^*DA/DA*^ BMDMs, which allowed us to limit expression of cFLIP without the potential confounding effects of inducing programmed necrosis. Importantly, IL-12 expression was similar in *Casp8* ^*DA/DA*^ and *Ripk3* ^*-/-*^ *Casp8* ^*DA/DA*^ BMDMs (Fig 7A and 7B). As expected, untreated cells expressed very low levels of the long and short forms of cFLIP (cFLIP_L_ and cFLIP_S_), and expression of both isoforms increased in response to TLR stimulation (Fig 7C). Critically, we observed significantly reduced levels of both cFLIP_L_ and cFLIP_S_ protein, but not caspase-8 itself, in the cFLIP siRNA-treated compared with control scramble (scr) siRNA-treated cells following LPS or CpG treatment (Fig 7C and 7D). Notably, although knockdown of cFLIP was not complete, it resulted in significantly lower levels of IL-12 secretion as well as reduced frequency of IL-12 positive cells relative to cells treated with scr siRNA (Fig 7D and 7E). Altogether, these data implicate cFLIP as an important regulator of caspase-8-dependent expression of inflammatory cytokines downstream of TLR signaling, and suggest that cFLIP and caspase-8 function together to promote inflammatory gene expression during sensing of microbial infection by TLRs.

## Discussion

Caspase-8 is a central regulator of cell fate decisions in the context of bacterial infection and inflammatory stimuli. The precise nature of the interactions between caspase-8 and a number of signaling and adapter proteins, such as cFLIP, RIPK1, RIPK3 and FADD, in the context of specific extracellular cues, determines whether the cell undergoes apoptosis, RIPK3-dependent programmed necrosis, or initiates inflammatory gene expression. Infection by the bacterial pathogen *Yersinia* induces innate immune cells to undergo caspase-8-mediated apoptosis, and it has been suggested that apoptosis of bacteria-infected cells promotes immune defense against infection [33, 61, 62]. However, whether caspase-8 controls anti-bacterial immune defense by regulating cell death or control of inflammatory cytokine production, as well as how caspase-8 controls inflammatory cytokine production during bacterial infection, remain unclear.

Germline mutations in human caspase-8 or its key adapter FADD cause a primary immunodeficiency associated with severe recurrent bacterial infections, encephalopathy and hepatopathy [10, 12]. While caspase-8 has been proposed to regulate NF-κB signaling in lymphocytes following antigen receptor activation [13, 31, 63], *Ripk3* ^*-/-*^ *Casp8* ^*-/-*^ T cells do not exhibit defects in NF-κB activation or antigen-specific IFNγ production [14, 15, 23, 64]. This suggested that caspase-8 regulates T cell responses by limiting RIPK3-dependent necrosis rather than controlling T cell activation itself. Nevertheless, several recent studies examining macrophages and dendritic cells from *Ripk3* ^*-/-*^ *Casp8* ^*-/-*^ animals have linked caspase-8 to the production of innate cytokines as well as to inflammasome activation [13, 23, 28, 29, 31, 64–69]. Interestingly, caspase-8 has a non-enzymatic, scaffolding, role in NLRP3 inflammasome activation [43]. Recent studies have come to different conclusions about the contribution of caspase-8 to direct activation of the IKK complex and IκB degradation in innate cells [23, 29]. Moreover, while caspase-8 mediates apoptosis and prevents RIPK3-necrosis downstream of the TLR4- or TLR3-TRIF axes [3–5, 52, 70], the extent to which caspase-8 regulates the program of TLR-induced gene expression has not been defined.

**Fig 8:**
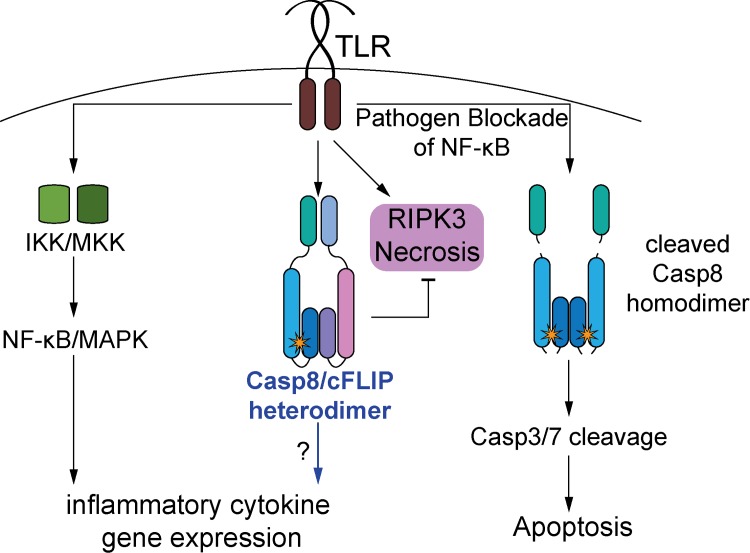
Model for the role of caspase-8 in inflammatory cytokine gene expression. TLR stimulation activates signaling complexes that mediate NF-κB and MAPK activation, which is required for induction of inflammatory cytokine gene expression. TLR stimulation also has the potential to induce programmed necrosis via RIPK3, or apoptosis via activation of caspase-8. Cleaved caspase-8 homodimers mediate apoptosis, whereas caspase-8/cFLIP heterodimers normally protect TLR-stimulated cells from programmed necrosis. Our data support a model whereby the caspase-8/cFLIP heterodimer also plays an important role in mediating inflammatory cytokine gene expression.

Here, we report a pleiotropic role for caspase-8 in the control of gene expression downstream of *Yersinia* and *Salmonella* infection, as well as all TLRs that we tested, including those that signal through MyD88. We also find that while caspase-8 enzymatic activity is necessary, caspase-8 autoprocessing is dispensable for its function in regulating inflammatory gene expression. As uncleaved caspase-8 acts in an enzymatically active complex with its homolog cFLIP, our findings support a model (Fig 8), whereby the enzymatic activity of a caspase-8/cFLIP complex promotes TLR-induced inflammatory gene expression.

Precisely how caspase-8 might be coupled to MyD88 downstream of TLR signaling is currently unclear, but both FADD and TRADD can associate with MyD88 through their death domains, providing a potential mechanism by which this could occur [71–73]. We nevertheless have not been able to detect endogenous caspase-8 at MyDDosomes in BMDMs, suggesting that if this interaction occurs it is either transient or unstable. It is possible that caspase-8 contributes to other innate signaling pathways, such as the NOD1/NOD2-RIPK2 pathway that detects the cytosolic presence of bacterial cell wall components. However, stimulation of macrophages with NOD2 ligands alone does not induce inflammatory cytokines; rather, NOD2 synergizes with TLR stimulation to enhance cytokine production [74–76]. A potential role for caspase-8 in facilitating this synergy is possible, but is difficult to distinguish from direct effects of caspase-8 on TLR signaling.

Caspase-8 regulates a critical subset of LPS-induced genes that individually are known to have key roles in inflammation and anti-microbial immune defense. Notably, IL-12, IL-6, pro-IL-1α, pro-IL-1β, as well as multiple chemokine genes were among the LPS-induced genes in cluster 2 that required caspase-8 for optimal induction. The 527 genes in both cluster 1 and 2 whose expression was significantly impacted by caspase-8 deletion collectively constitute 8.3% of total LPS-responsive genes in WT cells. Interestingly, cluster 1 genes were upregulated in the absence of caspase-8 relative to control B6 or *Ripk3* ^*-/-*^ cells. GO analysis of the genes in cluster 1 did not reveal significant enrichment for any specific biological pathways or terms. However, it is possible that elevated expression of some of these genes could play a negative feedback role in regulating the genes in cluster 2. Mechanistically, our data also demonstrate that caspase-8 enzymatic activity, most likely in the context of a caspase-8/cFLIP heterodimer, plays a key role in the induction of gene expression independently of apoptotic caspases or any other effects on cell death signaling. The precise targets of this activity remain to be defined, but do not involve the known apoptotic substrates caspase-3 and -7. Moreover, our findings that the gene expression defect of caspase-8-deficient cells is cell-intrinsic exclude a model in which the diminished cytokine production by caspase-8-deficient cells results from reduced release of intracellular alarmins. The profound loss of anti-microbial immune defense and severe susceptibility to bacterial infection in caspase-8-deficient animals is therefore likely the result of compound defects in antimicrobial cytokine production, rather than deficient apoptosis per-se.

It is possible that some of the effects we observe are the result of the dual deletion of RIPK3 and caspase-8. RIPK3 has been implicated in inflammatory responses independent of cell death, as RIPK3 itself can promote IL-1β expression in dendritic cells via induction of mitochondrial ROS [69]. Similarly, conditional deletion of caspase-8 in dendritic cells resulted in elevated production of IL-1β due to RIPK3-dependent activation of the NLRP3 inflammasome [77], and led to systemic autoimmunity [78]. Caspase-8 and RIPK3 may play cell type-specific roles in regulating inflammatory gene expression. However, the increased inflammatory responses observed in the setting of conditional deletion of caspase-8 or FADD *in vivo* are likely due to de-repression of RIPK3-dependent necrosis, because they are abrogated in a RIPK3-deficient setting [17, 20, 79]. We did not observe a contribution of RIPK3 to the caspase-8-dependent gene expression program, in that loss of RIPK3 alone did not substantially affect expression of pro-IL-1β or other inflammatory cytokines, either *in vitro* in BMDMs, or *in vivo* in monocytes or neutrophils in response to bacterial infection. While this manuscript was under review, the kinase activities of RIPK1 and RIPK3 were found to control inflammatory cytokine production in response to TLR4 stimulation via recruitment of Erk1/2 to the necrosome platform [80]. Interestingly, in BMDMs, this was observed only in the presence of caspase inhibitors, which is consistent with our observation that blockade of caspase activity in cells that are protected from programmed necrosis abrogates inflammatory gene expression.

Additional evidence further supports a role for caspase-8 in regulating gene expression independent of RIPK3: first, *Mlkl* ^*-/-*^ *Casp8* ^*-/-*^ mice have a significant defect in production of pro-inflammatory cytokines in response to multiple TLR agonists, despite having functional RIPK3. Second, treatment of RIPK3-sufficient B6 or *Ripk3* ^*-/-*^ BMDMs with a caspase-8 selective inhibitor, or the pan-caspase inhibitor QVD-oph, which do not potentiate programmed necrosis, also significantly reduced cytokine production in response to TLR stimulation. Additionally, reduced inflammatory gene expression in *Ripk3* ^*-/-*^ *Casp8* ^*-/-*^ cells is not due to a developmental defect in these cells because acute inhibition of caspase activity in *Ripk3* ^*-/-*^ cells was sufficient to inhibit TLR-induced gene expression.

The role of caspase-8 as a key modulator of gene expression makes it functionally analogous to RIPK1, which is also a central regulator of apoptosis, necrosis and gene expression [8]. It is possible that like RIPK1, distinct post-translational modifications or recruitment to distinct complexes mediate the switch between caspase-8-dependent apoptosis and gene expression. Caspase-8 may regulate gene expression through a RIPK1-containing complex distinct from complex II. However, since RIPK1 plays a central scaffolding role in receptor-proximal activation of the IKK complex, the effect of RIPK1 deficiency on TLR-induced gene expression is likely more profound than that of caspase-8.

Like TLR signaling, TNFR activation can trigger survival and cytokine production, apoptosis, or necrosis [7, 8]. Caspase-8 controls cell death in response to both TLR and TNFR signaling pathways. Nevertheless, the common chemokine genes *Cxcl2* and *Ccl22* that are induced by both LPS and TNF in BMDMs, required caspase-8 for maximal induction in response to LPS but not TNF. Whether caspase-8 contributes to TNFR-dependent gene expression in other cell types remains to be determined. Furthermore, although caspase-8-deficient BMDMs had a defect in their ability to respond to infection by gram-negative bacteria as well as multiple TLR agonists, *Ripk3* ^*-/-*^ *Casp8* ^*-/-*^ BMDMs produced equivalent levels of cytokines in response to Sendai virus infection, which engages the cytosolic PRRs RIG-I and MDA5. Our findings suggest that macrophages do not require caspase-8 for induction of inflammatory cytokines by cytosolic nucleic acid sensors. Thus, while caspase-8 regulates apoptosis and programmed necrosis downstream of multiple receptors, in macrophages, caspase-8 specifically controls gene expression in response to TLR signaling.

The defect in production of IL-6, TNF, IL-12p40 and proIL-1β by *Ripk3* ^*-/-*^ *Casp8* ^*-/-*^ cells in response to MyD88-dependent TLR agonists such as CpG and Pam3CSK4 could be due to the recently reported role of TRIF participating in signaling downstream of classical MyD88-dependent TLRs, as was suggested in the case of TLR2 [81]. However, TRIF does not contribute to TLR2-dependent induction of IL-6 or TNF [81], which are reduced in caspase-8-deficient cells in response to the TLR2 ligand Pam3CSK4. Therefore, caspase-8 may participate at a distal step of TLR signaling in a manner that affects both MyD88- and TRIF-dependent gene expression. We did not observe caspase-8-dependent regulation of receptor-proximal signaling events, such as Myddosome formation, IκBα degradation, or activation of the MAPK p38. Moreover, p65 was recruited equivalently to promoters of caspase-8-dependent genes in *Ripk3* ^*-/-*^ *Casp8* ^*-/-*^ cells, suggesting that nuclear localization of p65 is not affected by caspase-8 deficiency. These observations, along with the finding that IκBα resynthesis, which depends on NF-κB itself, was unaffected by caspase-8 deficiency, are consistent with our transcriptional profiling studies indicating that over 90% of LPS-induced gene expression remains unaffected in caspase-8-deficient cells.

It is currently unclear why caspase-8 plays a role in inflammatory cytokine expression by TLRs, but not IL-1R, which also signals through MyD88, or TNFR, which engages caspase-8 for apoptosis. However, differences in the response of macrophages to these stimuli are well established. For example, inflammatory cytokines are not induced in macrophages in response to TNF or IL-1β [38]. Recent studies revealed differences in the latent enhancers activated in macrophages in response to LPS, CpG, IL-1β, and TNF stimulation, suggesting that differences in signaling output exist between these stimuli despite their use of shared signaling components [40]. The engagement of caspase-8 by TLRs for transcriptional activation may indeed be one of the mediators of these differences. Differences in the stoichiometry or composition of specific downstream signaling components, such as IRAK-1 and IRAK-2 or particular TRAFs could potentially account for these differences. It is also possible that caspase-8 plays cell-type specific roles in response to stimulation of some receptors versus others. Identifying the potential caspase-8 substrates responsible for inflammatory cytokine induction may shed further light on this question.

Another unexpected finding here was that while *Casp8* ^*DA/DA*^ macrophages did not undergo apoptosis in response to *Yersinia* infection, they still undergo RIPK3-dependent necrosis, rather than being protected from death. This is in contrast to *Casp8* ^*DA/DA*^ macrophages treated with LPS, which do not have increased levels of programmed necrosis. This is likely due to the activity of YopJ in the context of *Yersinia* infection, which potently inhibits the IKK complex, and therefore likely impacts the ability of IKK complex to provide a transcription-independent prosurvival signal to RIPK1 [21]. Future studies will investigate the impact of switching cell death from apoptosis to necrosis in the context of *Yersinia* infection on host defense and inflammatory responses.

The precise target or targets of caspase-8/cFLIP activity that mediate this gene regulatory function remain to be identified. Nevertheless, our findings provide the first demonstration that enzymatic activity of caspase-8 plays a key cell-intrinsic role in TLR-dependent gene expression and control of *Yersinia* infection, independent of cell death. Together, these data provide new mechanistic insight into the non-apoptotic function of caspase-8 in innate immune defense, which may account for the severe susceptibility of mice and humans lacking caspase-8 to microbial infections.

## Materials and Methods

### Mice

C57BL/6.SJL mice were obtained from Jackson Laboratories. The *Ripk3* ^*-/-*^ *Casp8* ^*-/-*^ mice used in these studies were previously described [26] and provided by Doug Green (St. Jude Children’s Research Hospital). *Ripk3* ^*-/-*^ mice were provided by Vishva M. Dixit (Genentech). *Casp8* ^*f/-*^ mice were provided by Rasq Hakem (University Health Network). *Mlkl* ^*-/-*^ mice were provided by Warren Alexander (The Walter and Eliza Hall Institute of Medical Research). *Casp8* ^*+/-*^ mice that were used to generate the *Mlkl* ^*-/-*^ *Casp8* ^*-/-*^ mice were provided by Steve Heddrick (UCSD). *Casp3* ^*fl/fl*^Tie2-Cre^*+*^ and *Casp7* ^*-/-*^ mice were previously described [82, 83]. Six- to eight-week-old C57BL/6.SJL mice were lethally irradiated with 1100 rads and 2–5 x 10^6^ *Ripk3* ^*-/-*^, *Ripk3* ^*-/-*^ *Casp8* ^*-/-*^ or C57BL/6 (Jackson Laboratories) congenic bone marrow (BM) cells were transferred i.v. BM chimeras were allowed to reconstitute for eight to ten weeks. For other animal experiments, age- and sex-matched six-to-eight-week old mice were used.

### Generation of *Casp8* ^*DA/DA*^ mice

*Casp8* ^*DA/DA*^ mice were generated using the reagents and protocol described by Henao-Mejia and colleagues [84]. Briefly, single guide RNA (gRNA:ACAGAACCACACTTTAGAAG GTTTTAGAGCTAGAAATAGCAAGTTAAAATAAGGCTAGTCCGTTATCAACTTGAAAAAGTGGCACCGAGTCGGTGCTTTTTT) and Cas9 RNA were in vitro transcribed, purified and injected into B6xSJL F1 embryos with repair oligoDNA containing 50 base pairs of flanking regions on each side (T*T****GCC***TCATCATCTCACAAGAACTATATTCCGGATGAGGCAGAT). The complementary gRNA sequence is underlined, mutated base pairs are underlined and in italics, and the codon change for D387A is in boldface. Embryos were transferred to pseudopregnant females and pups were screened for mutagenesis using Surveyor Mutation Detection Kit (IDT). Founders were identified by cloning PCR products of tail DNA (primer F: TTCACTGGTTCAAAGTGCCC, primer R: ACTTTGCCAGAGCCTGAGGG) according to manufacturer’s instructions (M13 primers, TOPO TA Cloning Kit for Sequencing, ThermoFisher), followed by sequencing using T3 and T7 primers. Mice were backcrossed onto C57BL/6J for 5 generations until a minimum of 95% B6 was achieved by SNP analysis (Jax Genome Scanning Service). *Casp8* ^*DA/+*^ x *Casp8* ^*DA/+*^ mice were crossed and littermates were used for experiments.

### Animal infections

For *Yersinia* infections, mice were fasted for 12–16 hours and infected orally with 1–4 x 10^8^ *Yersinia* (32777). Mice were sacrificed and tissues were harvested on days 3 and 5 post-infection, as indicated. Bacterial load was determined by plating dilutions of tissue homogenates on LB+irgasan plates and single cell analysis was performed by flow cytometry. For *in vivo* Sendai virus infections, mice were anaesthetized using ketamine and infected with 10^4^ of SeV strain 52 (low defective viral genomes) per mouse [85]. Mice were weighed every one to two days. On days 3 and 10, lungs were harvested and homogenized in Trizol Reagent (Invitrogen/ThermoFisher) for RT-qPCR analysis.

### Flow cytometry

For animal studies, mesenteric lymph nodes were isolated and plated in complete-DMEM containing brefeldin A (Sigma) and monensin (BD) in a 37°C humidified incubator for 5 hrs. For BMDMs, cells were plated in 48- or 12-well suspension dishes and pre-treated with zVAD-fmk (100 μM) for 1 hr prior to PAMP stimulation, where indicated. BFA and monensin were added 1 hr later and samples were harvested for analysis 4–5 hrs later. BMDMs were harvested using PBS with EDTA (2mM) prior to staining. Cells were washed with PBS, stained for viability (Zombie Yellow, BioLegend) and then stained with the following antibodies from BioLegend: Ly6G (clone 1A-8 PE-Cy7), IL-6 (MP5-20F3 APC/FITC), F4/80 (clone BM8 Pacific Blue), BD Biosciences: CD45.1 (clone A20 APC-Cy7), CD45.2 (clone 104 FITC), MHCII (clone M5/114 BV650), ThermoFisher: CD11b (clone M1/70.15 PE-Texas Red), eBioscience: Ly6C (clone HK1.4 PerCPCy5.5), CD11c (clone N418 AF700), F480 (clone BM8 APC-eF780), TNF (clone MP6-XT22 eF450), IL-12p40 (clone C17.8 PE), proIL-1β (clone NJTEN3 APC/FITC), or cleaved caspase-3 (Asp 175, Cell Signaling Technologies). Inflammatory monocytes and neutrophils were gated as follows live CD45.1^+^2^+^/CD45.2^+^, CD11c^-^, CD11b^hi^, Ly6C^hi^ or Ly6G^+^. BMDMs were gated on Live/dead-, singlets, and in Fig 7 also CD11b^+^F480^+^. Resident peritoneal exudate cells (PECs) were isolated from naïve six- to eight-week-old sex-matched mice with cold PBS. PECs were incubated with LPS (10 ng/mL), BFA and monensin for 4hrs and large peritoneal macrophage responses were examined based on gating strategy as published by Ghosn et al. [86]. Surface staining was performed in FACS buffer (PBS with 1% BSA, 2mM EDTA) and sample fixation and permeabilization prior to intracellular staining were all performed according to manufacturer’s instructions (BD). Samples were run on an LSRFortessa and analyzed using FlowJo Treestar software.

### Cell culture and infection conditions

Bone marrow-derived macrophages (BMDMs) were grown as previously described [87] in a 37°C 5% CO_2_ humidified incubator in DMEM supplemented with 10% FBS, HEPES, sodium pyruvate (complete-DMEM) and 30% L929 supernatant for 7–9 days. 16–20 hrs prior to infection/treatment cells were re-plated into 96-, 48-, 24-, 12- or 6-well dishes in complete-DMEM containing 10% L929 supernatant. *Yersinia* were grown overnight with aeration in 2xYT broth at 26°C. *Salmonella* were grown overnight in LB medium at 37°C with aeration. Bacteria were washed three times with pre-warmed DMEM, added to the cells at an MOI of 20:1, and spun onto the cells at 1000 rpm for 5 min. Cells and bacteria were incubated at 37°C for 1 hr post-infection followed by addition of 100 μg/mL gentamicin. zVAD-fmk, QVD-oph, IETD-fmk or DEVD-fmk (100 μM, SM Biochemicals) were added 1 hr prior to treatment with LPS (100 ng/mL). GSK2399872A (GSK’872, 3 μm, GSK) was added 1 hr prior to infection where indicated. For *in vitro* Sendai virus infections, cells were washed twice with warm PBS, and infected with Sendai virus Cantell (high defective viral genomes) in a low volume of serum-free media as previously described [88].

### RT-qPCR

1x10^6^ BMDMs/well were plated in 6-well tissue culture-treated dishes 16 hrs before the experiment. Cells were treated with 10 ng/mL mIL-1β (eBioscience) for 2 hrs or with 10 ng/mL mTNFa (BioLegend) for 6hrs. For RNA stability experiments, BMDMs were stimulated with LPS (100 ng/mL) for 2 hrs, and then treated with Actinomycin D (Sigma, 5 μg/mL). Samples were lysed in Trizol Reagent (Invitrogen/ThermoFisher) and RNA was extracted using phenol/chloroform method. RNA was resuspended in RNAse-free water and cDNA synthesis was performed using High Capacity RNA to cDNA kit (ThermoFisher) as per manufacturer’s instructions. qPCR was run using Power Sybr Green Master Mix (ThermoFisher) on a QuantStudio Flex6000 (ThermoFisher). Primer sequences used are listed in S2 Table.

### RNA-seq

BMDMs were prepared as described above and 1x10^6^ BMDMs/well were plated in 6-well tissue culture-treated dishes and placed in a 5% CO_2_ 37°C incubator for 16 hours. BMDMs were stimulated with LPS (100 ng/mL) for 6 hrs. RNA was isolated using the RNeasy Mini Kit (Qiagen) and processed as per manufacturer’s instructions. mRNA-seq libraries were prepared using the TruSeq Stranded Total RNA LT Kit with Ribo-Zero Gold, according to the manufacturer’s instructions. Samples were run on Illumina NextSeq 500 to generate between 151 base-pair, paired-end reads with a Q30 score of ~80%, resulting in 25–50 million fragments/sample. All data processing and analyses were carried out using the R programming language (Version 3.2.2) and the RStudio interface (Version 0.99.489), as described previously [89] and can be reproduced using the supplementary code file. Briefly, raw fastq files were aligned to version 79 of mouse reference genome GRCm38 using the Subread aligner [90] in the RSubread package. BAM files were summarized to genes using the featureCounts algorithm [91]. Raw data is available on the Gene Expression Omnibus (GEO) (accession #GSE86922). Differentially expressed genes (≥1.5-fold and ≤5% false discovery rate) were identified by linear modeling and Bayesian statistics using the VOOM function [92] in the Limma package [93]. Gene Ontology (GO) was performed using the Database for Annotation, Visualization and Integration of Data (DAVID) [94, 95]. Gene Set Enrichment Analysis (GSEA) [96] was performed against the Molecular Signatures Database (MSigDB) [97] using the C2 canonical pathways collection.

### siRNA knockdown

BMDMs were seeded in 48-well tissue culture-treated dishes 16–20 hrs prior to transfection. The transfection protocol was modified from the “HiPerFect Transfection Reagent Handbook, Qiagen” for macrophage lines. Briefly, scramble or cFLIP pools of siRNA (25 nM, Dharmacon) were mixed with HiPerFect Transfection Reagent (Qiagen) in serum-free media, incubated at room temperature to allow RNA complex formation and added dropwise to cells cultured in an equal volume of complete DMEM (with serum, see above). Cells were incubated for 6 hrs under normal growth conditions. 2X volume of complete DMEM was carefully added and 18 hrs later, cell culture medium was replaced with complete DMEM containing 10% L929 supernatant. Cells were incubated for a further 24 hrs before treatment with PAMPs.

### ELISA

BMDMs were treated with 100 ng/mL *E*. *coli* LPS (Sigma), 1 μg/mL Pam3CSK4 (Invivogen), 1 μg/mL CpG (Invivogen), 50 μg/mL HMW Poly(I:C) (Invivogen). Release of proinflammatory cytokines was measured by enzyme-linked immunosorbent assay (ELISA) using capture and detection antibodies against IL-6 (BD), IL-12p40 (BD) or TNFα (BioLegend).

### LDH release

LDH release was quantified using the Cytotox96 Assay Kit (Promega) according to the manufacturer's instructions. Cytotoxicity was normalized to Triton (100%) and LDH release from untreated cells was used for background subtraction.

### Ethics statement

All animal studies were performed in compliance with the federal regulations set forth in the Animal Welfare Act (AWA), the recommendations in the Guide for the Care and Use of Laboratory Animals of the National Institutes of Health, and the guidelines of the University of Pennsylvania Institutional Animal Use and Care Committee. All protocols used in this study were approved by the Institutional Animal Care and Use Committee at the University of Pennsylvania (Multiple Project Assurance # A3709-01, Protocols #804523 and #805061).

## Supporting Information

S1 Fig Caspase-8 plays a cell-intrinsic role in inflammatory cytokine production during bacterial infection *in vivo*. (A) Schematic of mixed bone marrow chimera experimental set-up. Congenically marked B6 (white or black) or *Ripk3* ^*-/-*^ *Casp8* ^*-/-*^ (orange) bone marrow (BM) were injected at a 1:1 ratio into lethally-irradiated recipient B6.SJL mice. 8 weeks after reconstitution, chimeras were orally infected with *Yersinia* (4x10^8^/mouse) and immune responses were assayed at day 3 post-infection. (B) Representative flow plots of percentage of TNF-expressing Ly6C^hi^ inflammatory monocytes from B6.SJL:*R3* ^*-/-*^ *C8* ^*-/-*^, *R3* ^*-/-*^ *C8* ^*-/-*^ and B6:B6.SJL chimeras. Labels above plots indicate genotype of donor cells and labels to the right of the plots indicate genotype of chimeras. (C) Quantification of percentage of TNF^+^ monocytes from (B). Bars are color-coded to represent genotype of donor bone marrow (B6 = black, B6.SJL = white, *Ripk3* ^*-/-*^ *Casp8* ^*-/-*^ = orange). Brackets on the x-axis indicate genotype of mixed chimeras. (D) Degree of chimerism of inflammatory monocytes from infected mice analyzed in Fig 1B–1D. (E) Degree of chimerism of neutrophils from infected mice analyzed in Fig 1E and 1F. Bars are color-coded to represent genotype of donor bone marrow (B6 = black, *Ripk3* ^*-/-*^ = green, *Ripk3* ^*-/-*^ *Casp8* ^*-/-*^ = orange). (F) Bacterial loads/g tissue (CFU/g). Dotted lines represent limit of detection. Solid lines represent geometric means. *R3C8* = *Ripk3Casp8*. Representative of 4 independent *Yersinia* infection experiments performed with a minimum of 5–6 animals per group. * *p* < 0.05, ** *p* < 0.01, *** *p* < 0.001 by t-test.
(TIF)

S2 Fig Caspase-8 is not required for induction of NF-κB and MAPK signaling or p65 recruitment to inflammatory gene promoters. (A) B6 BMDMs were treated with LPS for indicated time points and lysates were immunoprecipitated with antibodies against MyD88 or control IgG and probed for MyD88, IRAK2 and caspase-8 (Casp8) by western analysis. Star represents background band. (B) B6, *Ripk3* ^*-/-*^ and *Ripk3* ^*-/-*^ *Casp8* ^*-/-*^ BMDMs were treated with LPS for 2hrs and lysates were immunoprecipitated with antibodies against MyD88 or control IgG and probed for MyD88 and IRAK2. (C) Kinetics of IκBα degradation and resynthesis post-LPS treatment was detected by western analysis. (D, E) Phosphorylation of AKT and p38 was probed by western post LPS-stimulation. Representative of two or more independent experiments. (F) Fold enrichment (% input in LPS-treated/% input untreated, see Methods) for p65 recruitment to promoters of *Ccl5*, *Cxcl2*, *Il12b*, *Il6*, *Tnf*, *Hbb-bs* and *Gapdh* in LPS-treated B6, *Ripk3* ^*-/-*^ and *Ripk3* ^*-/-*^ *Casp8* ^*-/-*^ BMDMs. *Hbb-bs* is not expressed in BMDMs (negative control) and *Gapdh* is a housekeeping gene. Samples were normalized to 5% input, error bars indicate +/- SD. Representative of 3 or more independently performed experiments for each panel.
(TIF)

S3 Fig Caspase-8 deficiency does not impact the stability of caspase-8-dependent mRNA. (A, B) B6, *Ripk3* ^*-/-*^ and *Ripk3* ^*-/-*^ *Casp8* ^*-/-*^ BMDMs were treated with LPS (100 ng/mL) for 2 hrs before addition of vehicle (DMSO) (A) or 5 μm actinomycin D (B). *Il12b*, *Il1b* and *Il6* mRNA was assayed by RT-qPCR at the indicated time points. Representative of two independent experiments.
(TIF)

S4 Fig Caspase-8-deficient cells exhibit wild-type levels of intracellular bacterial killing and responsiveness to cytokine stimulation. (A) Cells were treated as in Fig 3 and fold change of select genes from cluster 2 (refer to Fig 3E) (LPS vs UT). (B) BMDMs were treated with LPS (100 ng/mL) for 3 hrs, lysates were fractionated into cytoplasmic and nuclear extracts and probed for caspase-8, JunB, HDAC1 and β-tubulin by western blotting. (C) BMDMs were infected with *Salmonella* or *Yersinia* for 1hr, gentamycin was added, cells were lysed and CFUs were enumerated at the indicated time points. (D, E, F) RT-qPCR expression analysis of *Cxcl2* (D), *Ccl22* (E) and *Cxcl1* (F) from BMDMs treated with LPS (100 ng/mL) for 6 hrs (D, E, F), or TNF (10 ng/mL) for 6 hrs or as indicated (D, E) or IL-1β (10 ng/mL) for 2 and 6 hrs (F).
(TIF)

S5 Fig Caspase-8 is not required for cytokine production or clearance of Sendai infection. (A) B6, *Ripk3* ^*-/-*^ and *Ripk3* ^*-/-*^ *Casp8* ^*-/-*^ BMDMs were infected with Sendai virus (SeV) at an MOI of 10 for 6hrs. IL-6 and IL-12p40 release were measured by ELISA. (B) Transcript levels of *Ifnb*, *Il6* and *Il1b* from lungs of mock-infected (PBS) and SeV-infected (SeV) mice were assayed by RT-qPCR on day 3 post-infection. (C) Sendai virus nucleoprotein (SeV NP) levels in the lung were measured by RT-qPCR on day 3 (left) and day 10 (right) post-infection. (D) Weight loss in B6, *Ripk3* ^*-/-*^ and *Ripk3* ^*-/-*^ *Casp8* ^*-/-*^ mice that were infected intranasally with Sendai virus 52 (SeV). Representative of three independent experiments.
(TIF)

S6 Fig Control of TLR-induced gene expression by caspase-8 is independent of caspases-3/7. BMDMs of indicated genotypes were left unstimulated or treated with LPS (100 ng/mL) for 5 hrs. (A) Representative flow plots of IL-1β production as measured by flow cytometry. (B) Summary data of (A) and IL-12p40 release measured in BMDMs 5 hrs after LPS treatment. Representative of two independent experiments. (C) Expression of IL-6 and TNF in the presence of caspase-3/7 selective inhibitor DEVD-fmk (100 μM).
(TIF)

S1 Text Supporting experimental procedures and supplemental references. (DOCX)

S2 Text Supplementary code file used for analysis of RNASeq data. (PDF)

S1 Table Analysis of gene expression of LPS-responsive genes in B6, *Ripk3* ^*-/-*^ and *Ripk3* ^*-/-*^ *Casp8* ^*-/-*^ BMDMs. Reads per kilobase per million mapped reads (rpkm) log2 transformed, filtered and normalized (see also S2 Text, Supplementary Code File) of 6379 LPS-responsive genes identified in B6 BMDMs (worksheet 1). Differentially regulated genes between B6 and *Ripk3* ^*-/-*^ *Casp8* ^*-/-*^ BMDMs (Cluster 1 and 2 of Fig 3C, worksheet 2). Fold-change and Log (Fold-change) values comparing B6 and *Ripk3* ^*-/-*^ *Casp8* ^*-/-*^ or *Ripk3* ^*-/-*^ and *Ripk3* ^*-/-*^ *Casp8* ^*-/-*^ for Cluster 1 (worksheet 3) or Cluster 2 (worksheet 4). Expression of genes listed in Fig 3E (worksheet 5).
(XLSX)

S2 Table List of primer sequences used for ChIP and RT-qPCR analysis. Reference numbering refers to Supplemental References in S1 Text.
(DOCX)
