# Supplementary figures and images for: Activity of Uncleaved Caspase-8 Controls Anti-bacterial Immune Defense and TLR-Induced Cytokine Production Independent of Cell Death
Source: PLoS Pathog. 2016 Oct 13;12(10):e1005910. doi: 10.1371/journal.ppat.1005910 (PMC5063320; doi:10.1371/journal.ppat.1005910)

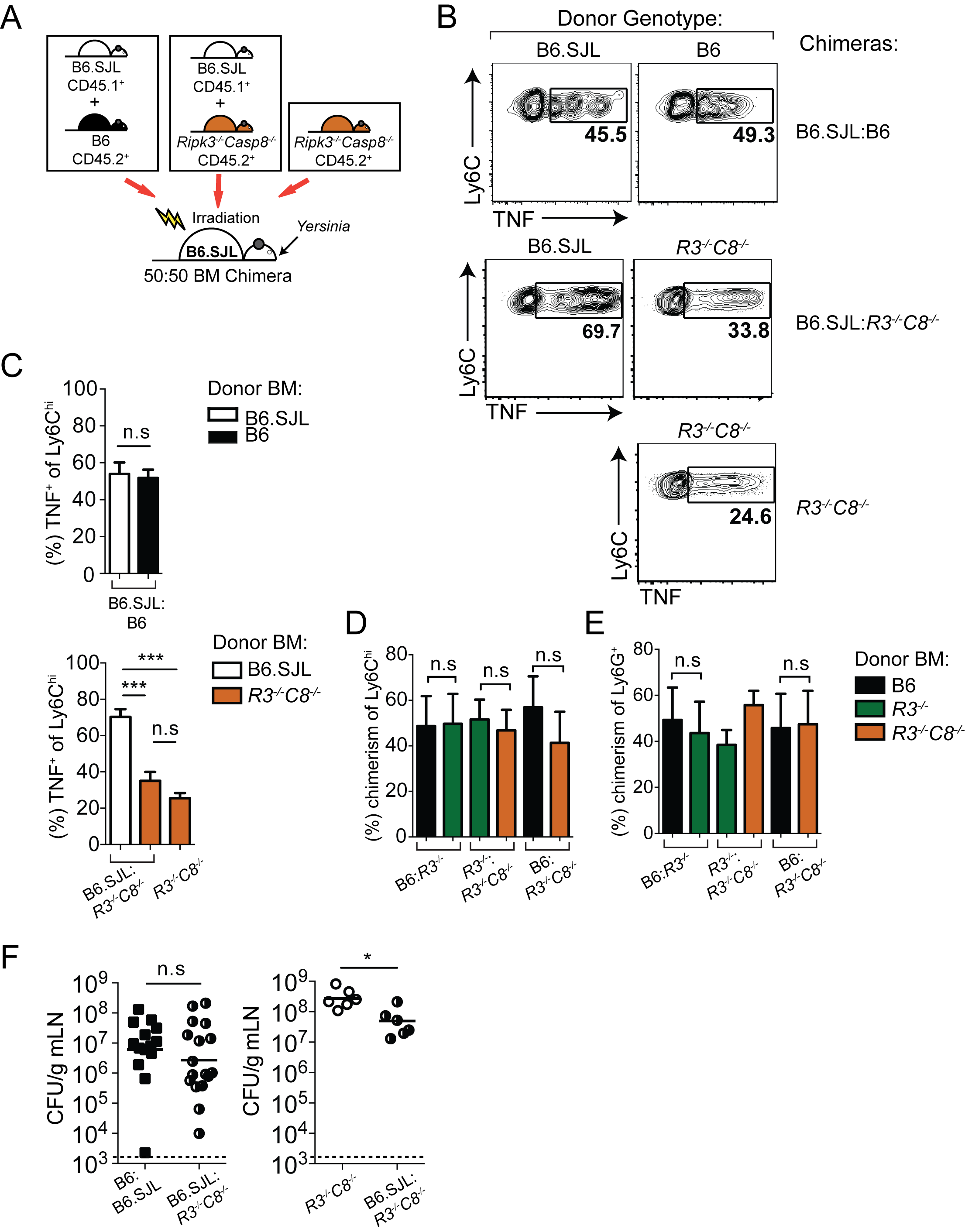

Supplement: S1 Fig — (A) Schematic of mixed bone marrow chimera experimental set-up. Congenically marked B6 (white or black) or Ripk3 -/- Casp8 -/- (orange) bone marrow (BM) were injected at a 1:1 ratio into lethally-irradiated recipient B6.SJL mice. 8 weeks after reconstitution, chimeras were orally infected with Yersinia (4x10^8^/mouse) and immune responses were assayed at day 3 post-infection. (B) Representative flow plots of percentage of TNF-expressing Ly6Chi inflammatory monocytes from B6.SJL:R3 -/- C8 -/-, R3 -/- C8 -/- and B6:B6.SJL chimeras. Labels above plots indicate genotype of donor cells and labels to the right of the plots indicate genotype of chimeras. (C) Quantification of percentage of TNF+ monocytes from (B). Bars are color-coded to represent genotype of donor bone marrow (B6 = black, B6.SJL = white, Ripk3 -/- Casp8 -/- = orange). Brackets on the x-axis indicate genotype of mixed chimeras. (D) Degree of chimerism of inflammatory monocytes from infected mice analyzed in Fig 1B–1D. (E) Degree of chimerism of neutrophils from infected mice analyzed in Fig 1E and 1F. Bars are color-coded to represent genotype of donor bone marrow (B6 = black, Ripk3 -/- = green, Ripk3 -/- Casp8 -/- = orange). (F) Bacterial loads/g tissue (CFU/g). Dotted lines represent limit of detection. Solid lines represent geometric means. R3C8 = Ripk3Casp8. Representative of 4 independent Yersinia infection experiments performed with a minimum of 5–6 animals per group. * p < 0.05, ** p < 0.01, *** p < 0.001 by t-test. (TIF) [file ppat.1005910.s001.tif]

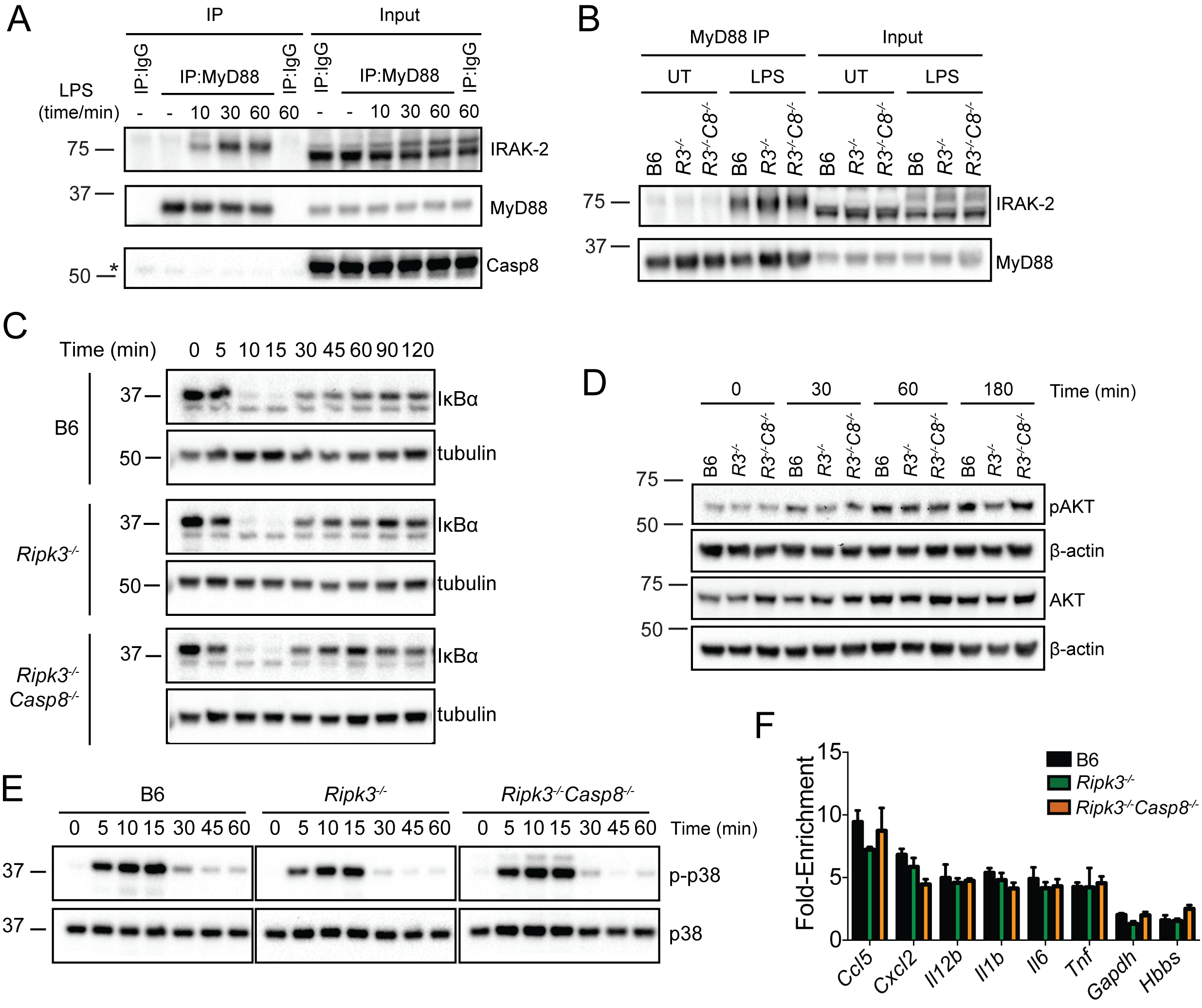

Supplement: S2 Fig — (A) B6 BMDMs were treated with LPS for indicated time points and lysates were immunoprecipitated with antibodies against MyD88 or control IgG and probed for MyD88, IRAK2 and caspase-8 (Casp8) by western analysis. Star represents background band. (B) B6, Ripk3 -/- and Ripk3 -/- Casp8 -/- BMDMs were treated with LPS for 2hrs and lysates were immunoprecipitated with antibodies against MyD88 or control IgG and probed for MyD88 and IRAK2. (C) Kinetics of IκBα degradation and resynthesis post-LPS treatment was detected by western analysis. (D, E) Phosphorylation of AKT and p38 was probed by western post LPS-stimulation. Representative of two or more independent experiments. (F) Fold enrichment (% input in LPS-treated/% input untreated, see Methods) for p65 recruitment to promoters of Ccl5, Cxcl2, Il12b, Il6, Tnf, Hbb-bs and Gapdh in LPS-treated B6, Ripk3 -/- and Ripk3 -/- Casp8 -/- BMDMs. Hbb-bs is not expressed in BMDMs (negative control) and Gapdh is a housekeeping gene. Samples were normalized to 5% input, error bars indicate +/- SD. Representative of 3 or more independently performed experiments for each panel. (TIF) [file ppat.1005910.s002.tif]

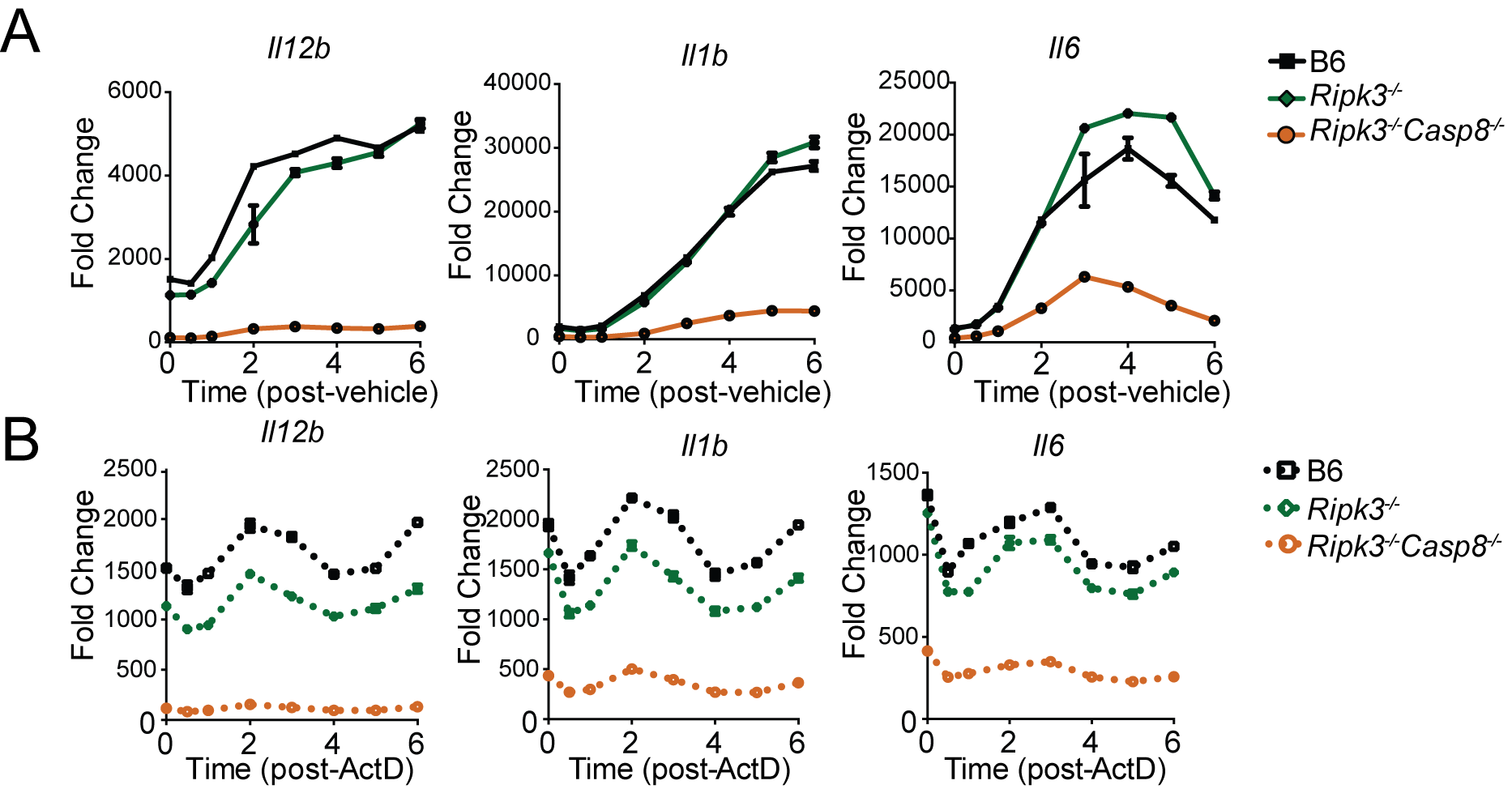

Supplement: S3 Fig — (A, B) B6, Ripk3 -/- and Ripk3 -/- Casp8 -/- BMDMs were treated with LPS (100 ng/mL) for 2 hrs before addition of vehicle (DMSO) (A) or 5 μm actinomycin D (B). Il12b, Il1b and Il6 mRNA was assayed by RT-qPCR at the indicated time points. Representative of two independent experiments. (TIF) [file ppat.1005910.s003.tif]

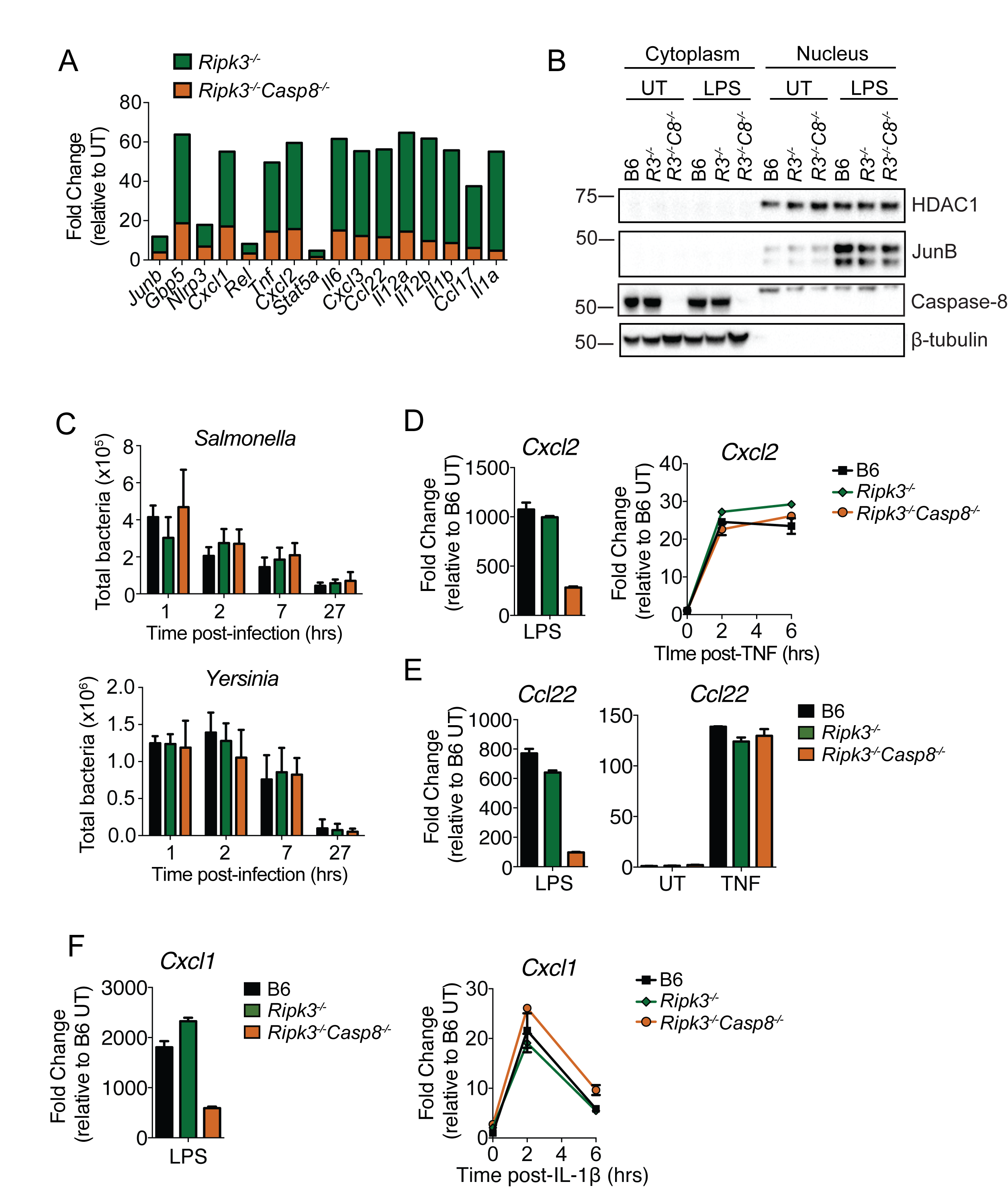

Supplement: S4 Fig — (A) Cells were treated as in Fig 3 and fold change of select genes from cluster 2 (refer to Fig 3E) (LPS vs UT). (B) BMDMs were treated with LPS (100 ng/mL) for 3 hrs, lysates were fractionated into cytoplasmic and nuclear extracts and probed for caspase-8, JunB, HDAC1 and β-tubulin by western blotting. (C) BMDMs were infected with Salmonella or Yersinia for 1hr, gentamycin was added, cells were lysed and CFUs were enumerated at the indicated time points. (D, E, F) RT-qPCR expression analysis of Cxcl2 (D), Ccl22 (E) and Cxcl1 (F) from BMDMs treated with LPS (100 ng/mL) for 6 hrs (D, E, F), or TNF (10 ng/mL) for 6 hrs or as indicated (D, E) or IL-1β (10 ng/mL) for 2 and 6 hrs (F). (TIF) [file ppat.1005910.s004.tif]

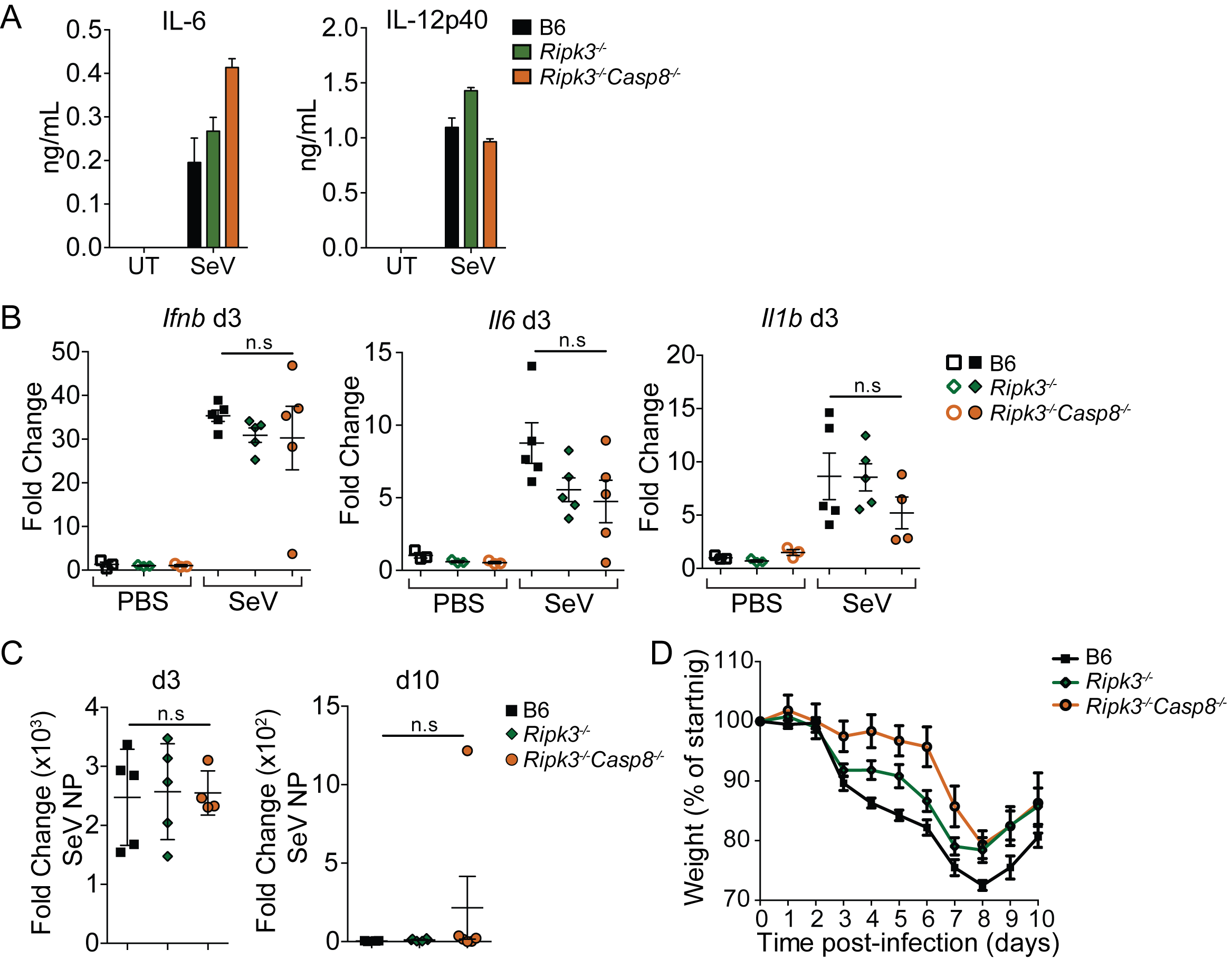

Supplement: S5 Fig — (A) B6, Ripk3 -/- and Ripk3 -/- Casp8 -/- BMDMs were infected with Sendai virus (SeV) at an MOI of 10 for 6hrs. IL-6 and IL-12p40 release were measured by ELISA. (B) Transcript levels of Ifnb, Il6 and Il1b from lungs of mock-infected (PBS) and SeV-infected (SeV) mice were assayed by RT-qPCR on day 3 post-infection. (C) Sendai virus nucleoprotein (SeV NP) levels in the lung were measured by RT-qPCR on day 3 (left) and day 10 (right) post-infection. (D) Weight loss in B6, Ripk3 -/- and Ripk3 -/- Casp8 -/- mice that were infected intranasally with Sendai virus 52 (SeV). Representative of three independent experiments. (TIF) [file ppat.1005910.s005.tif]

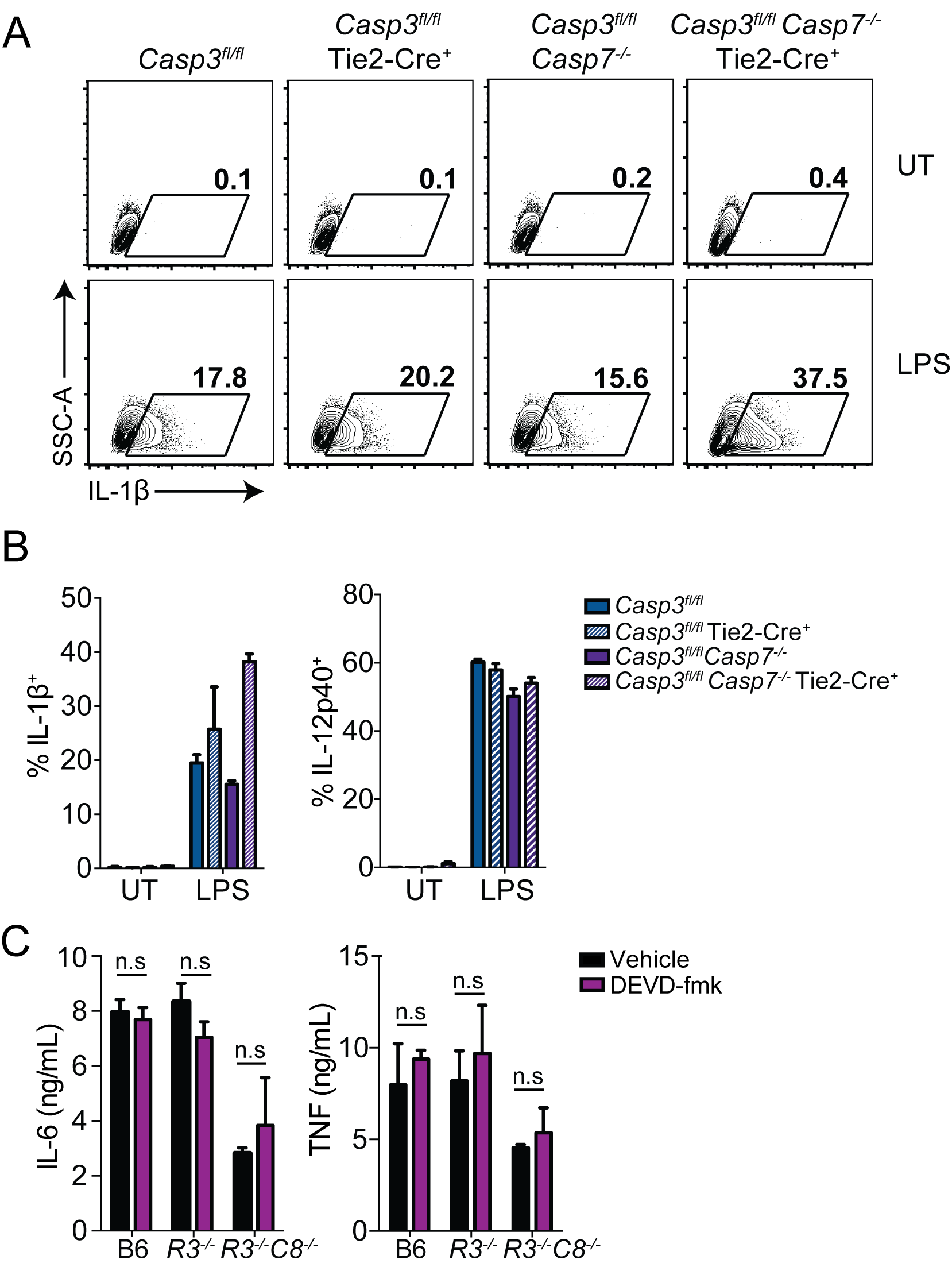

Supplement: S6 Fig — BMDMs of indicated genotypes were left unstimulated or treated with LPS (100 ng/mL) for 5 hrs. (A) Representative flow plots of IL-1β production as measured by flow cytometry. (B) Summary data of (A) and IL-12p40 release measured in BMDMs 5 hrs after LPS treatment. Representative of two independent experiments. (C) Expression of IL-6 and TNF in the presence of caspase-3/7 selective inhibitor DEVD-fmk (100 μM). (TIF) [file ppat.1005910.s006.tif]
