# Supplementary material for: Activity of Uncleaved Caspase-8 Controls Anti-bacterial Immune Defense and TLR-Induced Cytokine Production Independent of Cell Death
Source: PLoS Pathog. 2016 Oct 13;12(10):e1005910. doi: 10.1371/journal.ppat.1005910 (PMC5063320; doi:10.1371/journal.ppat.1005910)
Supplement: S1 Text — (DOCX) [file ppat.1005910.s007.docx]

**Supplemental Experimental Procedures**

**Western Blotting**

Whole cell lysate western blotting was adapted from [1]. Briefly, cells were lysed in 20 mM HEPES, 150 mM NaCl, 10% glycerol, 1% Triton X-100, 1 mM EDTA, 1 mM NaF and 1 mM activated Na_3_VO_4_. Lysates were mixed with protein loading buffer, boiled, centrifuged, and 20% of the total cell lysate loaded onto 4%–12% NuPAGE gels (Invitrogen). Proteins were transferred to PVDF membrane (Millipore) and blotted with rabbit anti-mouse MAPK and phospho-MAPK antibodies (all from Cell Signaling Technologies), rat anti-mouse caspase-8 (clone 1G12, ALX 804-447-C100, Enzo Life Sciences), mouse anti-mouse β-actin (Sigma), mouse anti-mouse β-tubulin (Sigma), anti-IRAK2 (ProSci 3595 rabbit anti-mouse), anti-HDAC1 (Cell Signaling Technologies), anti-JunB (Cell Signaling Technologies 3753S). Secondary antibodies were goat anti-rabbit, goat anti-rat (Jackson Immunoresearch) or horse anti-mouse HRP (Cell Signaling Technology).

**Immunoprecipitations**

Myddosome isolation assays were adapted from [2]. Briefly, BMDMs were treated with LPS (100 ng/mL) and harvested in cold PBS. 4x10^6^ cells were used per IP condition. Cells were lysed in 1% NP-40 (IGEPAL^®^ CA-630 Sigma), 50 mM Tris-HCl (pH 7.4), 150 mM NaCl, 10% glycerol, and protease/phosphatase inhibitors (Roche) for 20 minutes on ice and spun at high speed for 20 minutes. 1/7 of lysates was aliquoted for inputs, the remaining lysate was incubated at 4°C with 1 μg anti-MyD88 (R&D LifeSciences, AF3109 goat anti-mouse) or control goat IgG (R&D Normal Goat IgG Control, AB-108-C). The following day, 50 μL of protein G Dynabeads (ThermoFisher) was added for 1 hr at 4°C. Beads were washed 3 times with lysis buffer, proteins were extracted by adding Laemmli buffer, boiled, electrophoresed, and immunoblotted with the indicated antibodies using standard conditions.

**Nuclear Extractions**

5x10^6^ cells per condition were treated as described and harvested with cold PBS. Pellets were resuspended in 100 μL cold NAR A (10 mM HEPES pH 7.9, 10 mM KCl, 0.1 mM EDTA pH 8, protease/phosphatase inhibitors, 1mM DTT, 1mM β-glycerophasphate and 1mM NaF) and incubated on ice for 20 minutes. 10 μL 1% NP-40 was added and lysates were incubated at room temperature for 2-5 minutes. Lysates were vortexed for 10-30 seconds, centrifuged for 1.5 min at 6000 rpm. Supernatants containing the crude cytoplasmic extract were transferred to new microcentrifuge tubes. Cytoplasmic extracts were spun for 60 minutes at full speed at 4°C and supernatants containing purified cytoplasmic extracts were recovered. Nuclear pellets from first wash were washed four times in 100 μL cold NAR A. Pellets were resuspended in 50 μL NAR C (20 mM HEPES pH 7.9, 0.4 M NaCl pH 8, 1 mM EDTA pH 8, protease/phosphatase inhibitors, 1 mM DTT, 1 mM β-glycerophasphate and 1 mM NaF). Tubes were vortexed at 4°C for 1 hr at full speed. Supernatants containing nuclear extracts were recovered after max speed spin for 20 minutes. All lysates were quantified, run on SDS-PAGE and analyzed by western blotting.

**Chromatin Immunoprecipitation**

Bone marrow-derived macrophages (BMDMs) were stimulated with LPS (50 ng/mL, Sigma) for 2 hrs. Cells were cross-linked with disuccinimidyl glutarate (2 mM, ThermoFisher) for 30 min, then formaldehyde (1%, Sigma) for 10 min and quenched with glycine (0.125 M) for 5 min. Nuclear lysis was performed with the truChIP Chromatin Shearing Reagent KIT (Covaris) and nuclei were sonicated using the Covaris S220 sonicator. Chromatin was immunoprecipitated using anti-p65 antibody sc-372X (Santa Cruz) and protein G agarose beads (Millipore). All samples were column purified with the QIAquick PCR purification Kit (Qiagen). qPCR was performed using SYBER Green PCR Master Mix (ThermoFisher). Primer pairs were designed to amplify 80-250 bp fragments of the promoter region of each gene (Table S2) and melt curve analysis was used to confirm the amplification of unique products. All samples were normalized to a 5% input control and fold enrichment calculated using percent enrichment of treated over percent enrichment for untreated samples.

**Supplemental References**

1. Brodsky IE, Palm NW, Sadanand S, Ryndak MB, Sutterwala FS, Flavell RA, et al. A Yersinia effector protein promotes virulence by preventing inflammasome recognition of the type III secretion system. Cell Host Microbe. 2010;7(5):376-87. doi: 10.1016/j.chom.2010.04.009. PubMed PMID: 20478539; PubMed Central PMCID: PMCPMC2883865.

2. Bonham KS, Orzalli MH, Hayashi K, Wolf AI, Glanemann C, Weninger W, et al. A promiscuous lipid-binding protein diversifies the subcellular sites of toll-like receptor signal transduction. Cell. 2014;156(4):705-16. doi: 10.1016/j.cell.2014.01.019. PubMed PMID: 24529375; PubMed Central PMCID: PMCPMC3951743.

3. Ramirez-Carrozzi VR, Braas D, Bhatt DM, Cheng CS, Hong C, Doty KR, et al. A unifying model for the selective regulation of inducible transcription by CpG islands and nucleosome remodeling. Cell. 2009;138(1):114-28. doi: 10.1016/j.cell.2009.04.020. PubMed PMID: 19596239; PubMed Central PMCID: PMCPMC2712736.

4. Yount JS, Kraus TA, Horvath CM, Moran TM, Lopez CB. A novel role for viral-defective interfering particles in enhancing dendritic cell maturation. J Immunol. 2006;177(7):4503-13. PubMed PMID: 16982887.

5. Sun Y, Jain D, Koziol-White CJ, Genoyer E, Gilbert M, Tapia K, et al. Immunostimulatory Defective Viral Genomes from Respiratory Syncytial Virus Promote a Strong Innate Antiviral Response during Infection in Mice and Humans. PLoS Pathog. 2015;11(9):e1005122. doi: 10.1371/journal.ppat.1005122. PubMed PMID: 26336095; PubMed Central PMCID: PMCPMC4559413.

6. Tapia K, Kim WK, Sun Y, Mercado-Lopez X, Dunay E, Wise M, et al. Defective viral genomes arising in vivo provide critical danger signals for the triggering of lung antiviral immunity. PLoS Pathog. 2013;9(10):e1003703. doi: 10.1371/journal.ppat.1003703. PubMed PMID: 24204261; PubMed Central PMCID: PMCPMC3814336.
