# Supplementary material for: Activity of Uncleaved Caspase-8 Controls Anti-bacterial Immune Defense and TLR-Induced Cytokine Production Independent of Cell Death
Source: PLoS Pathog. 2016 Oct 13;12(10):e1005910. doi: 10.1371/journal.ppat.1005910 (PMC5063320; doi:10.1371/journal.ppat.1005910)
Supplement: S2 Text — (PDF) [file ppat.1005910.s008.pdf]

Supplementary Code File

Contents

Abstract . . . 1
R Packages . . . 1
Set-up and QC . . . 2
Exploratory Analysis . . . 3
Identification of Differentially Expressed Genes . . . 6

Abstract

This document contains all the code used to analyze the RNA-seq data in the manuscript entitled "Enzymatic activity of uncleaved caspase-8 tunes TLR-induced inflammatory gene expression." Briefly, B6, *Ripk3*$^{-/-}$ and *Ripk3*$^{-/-}$ *Casp8*$^{-/-}$ BMDMs were stimulated with LPS for 6hrs or left untreated. RNA was isolated using the RNeasy Mini Kit (Qiagen) and processed as per manufacturer's instructions. mRNA-seq libraries were prepared using the TruSeq Stranded Total RNA LT Kit with Ribo-Zero Gold, according to the manufacturer's instructions. Samples were run on Illumina NextSeq 500 to generate between 151 base-pair, paired-end reads with a Q30 score of ~80%, resulting in 25-50 million fragments/sample. All data processing and analyses were carried out using the R programming language (Version 3.2.2) and the RStudio interface (Version 0.99.489), and can be reproduced using this supplementary code file.

R Packages

These are the R/Bioconductor packages that were used for this analysis

```
library(Rsubread)
library(limma)
library(edgeR)
library(ShortRead)
library(org.Mm.eg.db)
library(AnnotationDbi)
library(WGCNA)
library(ggplot2)
library(reshape2)
library(dplyr)
library(ggvis)
library(Biobase)
library(RColorBrewer)
library(gplots)
```

This dynamic html summary report was compiled in Rmarkdown using the following packages:

```
library(rmarkdown)
library(knitr)
library(devtools)
```

Set-up and QC

To start off, I read in a file that describes the design of the study. This file sets treatment groups, replicates, sample names and more

```
targets <- readTargets("Igor_Ripk3_BMMC_studyDesign_RNAseq.txt", row.names=NULL)
targets.mod <- targets[1:12,]
myGroups <- factor(paste(targets.mod$genotype, targets.mod$treatment, sep="."))
sampleLabels <- paste(targets.mod$genotype, targets.mod$treatment, targets.mod$rep, sep=".")
design <- model.matrix(~0+myGroups)
colnames(design) <- levels(myGroups)
```

Then I read in my raw data. Fastq files were previously aligned to the mouse genome Mus_musculus.GRCm38 to generate BAM files. Reads were then aligned to exons and compiled in a digital gene expression (DGE) list.

```
load("DGElist")
myEnsemblIDs <- DGEList$genes$GeneID
```

Normalize unfiltered data based on mean-variance relationship and generate log2 counts per million (CPM) and reads per kilobase per million (RPKM)

```
normData.unfiltered <- voom(DGEList, design, plot=FALSE)
exprs.unfiltered <- normData.unfiltered$E
exprs.matrix.unfiltered <- as.matrix(exprs.unfiltered)
rpkm.unfiltered <- rpkm(DGEList, DGEList$genes$Length)
```

Filter data to only keep those genes that had >10 reads per million mapped reads in atleast two libraries.

```
cpm.matrix.filtered <- rowSums(cpm(DGEList) > 10) >= 2
DGEList.filtered <- DGEList[cpm.matrix.filtered,]
rpkm.filtered <- rpkm(DGEList.filtered, DGEList.filtered$genes$Length)
rpkm.filtered <- log2(rpkm.filtered + 1)
normData <- voom(DGEList.filtered, design, plot=FALSE)
exprs <- normData$E
exprs.matrix.filtered <- as.matrix(exprs)
```

Annotate expression data using the updated mouse database, insert EntrezIDs and filter out genes on X and Y chromosome as mixed-sex BMDMs were used.

```
columns(org.Mm.eg.db)
keytypes(org.Mm.eg.db)
myAnnot.unfiltered <- AnnotationDbi::select(org.Mm.eg.db, keys=rownames(exprs.matrix.unfiltered),
                       keytype="ENSEMBL", columns=c("ENTREZID", "GENENAME", "SYMBOL", "CHR"))
myAnnot.filtered <- AnnotationDbi::select(org.Mm.eg.db, keys=rownames(exprs.matrix.filtered),
                     keytype="ENSEMBL", columns=c("ENTREZID", "GENENAME", "SYMBOL", "CHR"))
resultTable.unfiltered <- merge(myAnnot.unfiltered, exprs.matrix.unfiltered, by.x="ENSEMBL", by.y=0)
resultTable.filtered <- merge(myAnnot.filtered, exprs.matrix.filtered, by.x="ENSEMBL", by.y=0)
head(resultTable.unfiltered)
dim(resultTable.unfiltered)
dim(resultTable.filtered)
```

```r
#add more appropriate sample names as column headers
colnames(resultTable.unfiltered) <- c("Ensembl", "Entrez", "Name", "Symbol", "Chr", sampleLabels)
colnames(resultTable.filtered) <- c("Ensembl", "Entrez", "Name", "Symbol", "Chr", sampleLabels)
#now write these annotated datasets out
write.table(resultTable.unfiltered, "normalizedUnfilteredRNAseq.txt", sep="\t", quote=FALSE)
write.table(resultTable.filtered, "normalizedFilteredRNAseq.txt", sep="\t", quote=FALSE)
head(resultTable.unfiltered)
head(resultTable.filtered)
#remove all genes on X and Y chromosomes using dplyr filter function as mixed sex BMDMs were used
class(resultTable.filtered)
dim(resultTable.filtered)
myDataX.filter <- resultTable.filtered %>%
  filter(Chr!="X")
head(myDataX.filter)
myDataXY.filter <- myDataX.filter %>%
  filter(Chr!="Y")
head(myDataXY.filter)
dim(myDataXY.filter)
write.table(myDataXY.filter, "normalizedFilteredXYRNAseq.txt", sep="\t", quote=FALSE)
```

Since RNA-seq may bring up multiple gene segments per gene ID, collapse expression data so that each gene corresponds to one set of values.

```r
dupFiltered <- unique(myDataXY.filter$Symbol)
myIDs <- rownames(myDataXY.filter)
mySymbols <- myDataXY.filter[,4]
head(myDataXY.filter)
resultTable <- myDataXY.filter[,-1:-5]
head(resultTable)
myCollapsed <- collapseRows(resultTable, mySymbols, myIDs, method = "MaxMean")
myCollapsed <- myCollapsed$datETcollapsed
write.table(myCollapsed, "Collapsed.txt", sep="\t", quote=FALSE)
dim(myCollapsed)
```

Exploratory Analysis

Here, I plot a cluster dendrogram that groups samples based on similarity

```r
sampleLabels <- paste(targets$genotype, targets$treatment, sep=".")
distance <- dist(t(exprs.matrix.filtered),method="maximum")
clusters <- hclust(distance, method = "complete")
plot(clusters, label = sampleLabels, hang = -1)
```

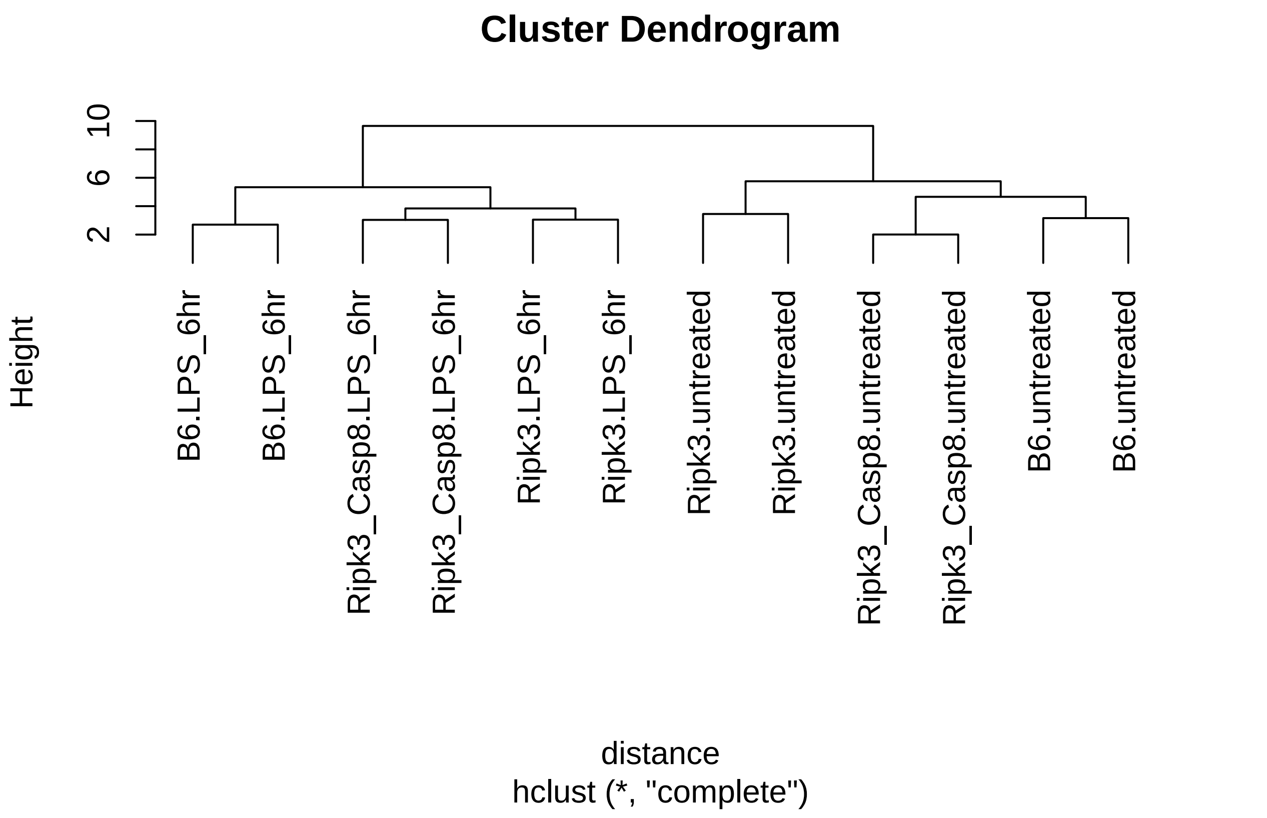

Figure 1: Samples group by genotype and treatment.

Principal component analysis of these data portrays variables that account for most of the variation in the data

Figure 2: LPS accounts for over 95% of the variance.

```
pca.res <- prcomp(t(exprs.matrix.filtered), scale.=F, retx=T)
ls(pca.res)
summary(pca.res)
head(pca.res$rotation)
head(pca.res$x)
plot(pca.res, las=1)
```

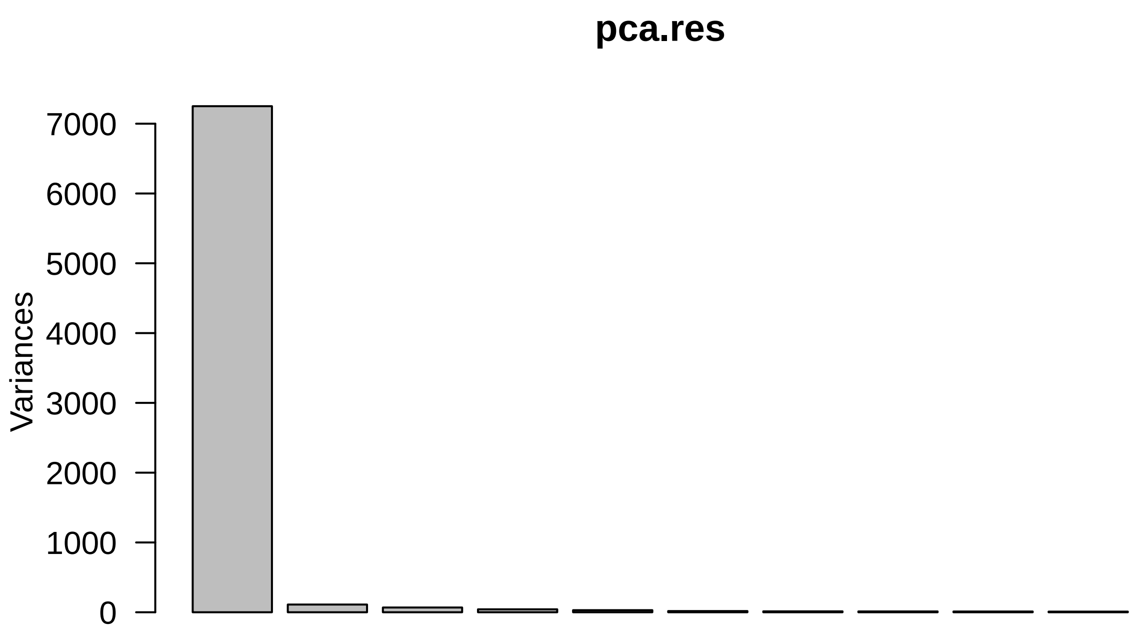


```
pc.var<-pca.res$sdev^2
pc.per<-round(pc.var/sum(pc.var)*100, 1)
pc.per
```

Now we can visualize how the samples contribute to PC1 and PC2

```
data.frame <- as.data.frame(pca.res$x)
ggplot(data.frame, aes(x=PC1, y=PC2, color=myGroups)) +
  geom_point(size=5) +
  scale_colour_manual(values=c("#000000", "#AAAAAA", "#E36B09", "#FFCC66", "#297A18", "#6EF054")) +
  theme(legend.position="right", legend.key=element_rect(fill=NA)) +
  #remove axis ticks and tick text
  theme(axis.ticks = element_blank(),axis.text = element_blank()) +
  #change background and gridlines
  theme(panel.background = element_rect(fill = NA, colour = "black"),
        panel.grid.major = element_line(colour = NA),
        panel.grid.minor = element_line(colour = NA))
```

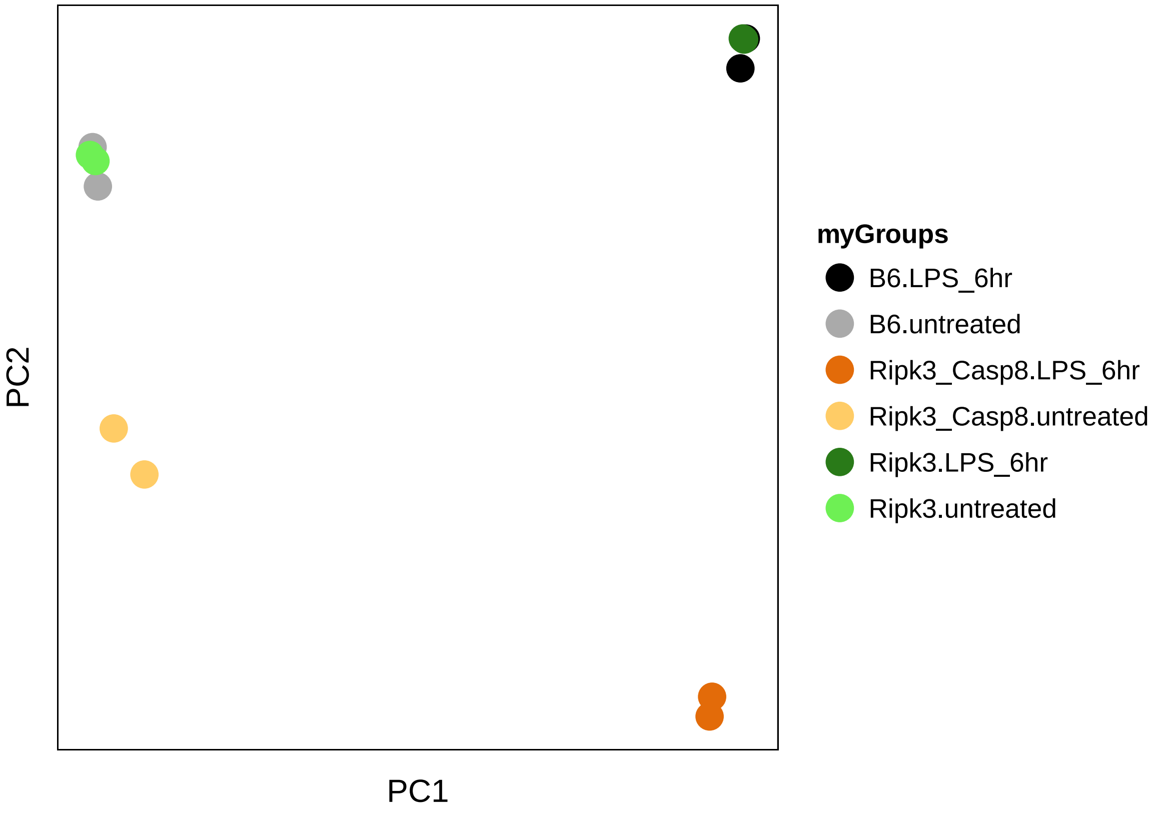

Figure 3: LPS-treatment contributes to the largest variation between samples (96%), where B6 and *Ripk3*-/- cluster together and are separated from the corresponding *Ripk3*-/- *Casp8*-/- samples.

Identification of Differentially Expressed Genes

I fit a linear model to the data and set up contrast matrices of the analyses I'm interested in.

```
fit <- lmFit(myCollapsed, design)
# set up a contrast matrix comparing UT and LPS in B6 BMDMs = "LPS-responsive genes"
contrast.matrix.UT.LPS.B6 <- makeContrasts(B6UT.v.B6LPS = B6.LPS_6hr - B6.untreated, levels=design)
# extract the linear model fit for the contrast matrix that you just defined above
fitsUT.LPS.B6 <- contrasts.fit(fit, contrast.matrix.UT.LPS.B6)
#get bayesian stats for your linear model fit
ebFitUT.LPS.B6 <- eBayes(fitsUT.LPS.B6)
```

Here, I pull out probeIDs from all genes in the Venn diagram and combine them with expression data into one file.

```
# use the 'decideTests' function to show Venn diagram for all diffexp genes
resultsUT.LPS.B6 <- decideTests(ebFitUT.LPS.B6, method="global",
                                adjust.method="BH", p.value=0.05, lfc=0.59)
diffProbesUT.LPS.B6 <- which(resultsUT.LPS.B6[,1] !=0)
myEset.ALL <- new("ExpressionSet", exprs = myCollapsed)
# retrieve expression data for the probes from above and export into excel table
diffDataUT.LPS.B6 <- myEset.ALL[resultsUT.LPS.B6[,1] !=0]
diffDataUT.LPS.B6 <- exprs(diffDataUT.LPS.B6)
write.table(cbind(diffProbesUT.LPS.B6, diffDataUT.LPS.B6),
            "DiffGenesUTvsLPSB6.xls", sep="\t", quote=FALSE)
```

Now, use LPS-responsive genes (diffDataUT.LPS.B6) as input gene list to determine which genes are caspase-8 and/or RIPK3-dependent.

```
fit <- lmFit(diffDataUT.LPS.B6, design)
# set up contrast matrices based on the pairwise comparisons of interest
contrast.matrix.LPS.ofB6LPSR3 <- makeContrasts(R3.v.B6 = Ripk3.LPS_6hr - B6.LPS_6hr, levels=design)
contrast.matrix.LPS.ofB6LPSR3C8 <- makeContrasts(R3C8.v.R3 = Ripk3_Casp8.LPS_6hr - Ripk3.LPS_6hr,
                                 R3C8.v.B6 = Ripk3_Casp8.LPS_6hr - B6.LPS_6hr, levels=design)

# extract the linear model fit for the contrast matrix that you just defined above
fitsLPS.ofB6LPSR3 <- contrasts.fit(fit, contrast.matrix.LPS.ofB6LPSR3)
fitsLPS.ofB6LPSR3C8 <- contrasts.fit(fit, contrast.matrix.LPS.ofB6LPSR3C8)

#get bayesian stats for your linear model fit
ebFitLPS.ofB6LPSR3 <- eBayes(fitsLPS.ofB6LPSR3)
ebFitLPS.ofB6LPSR3C8 <- eBayes(fitsLPS.ofB6LPSR3C8)

resultsLPS.ofB6LPSR3  <- decideTests(ebFitLPS.ofB6LPSR3, method="global",
                          adjust.method="BH", p.value=0.05, lfc=0.59)
resultsLPS.ofB6LPSR3C8  <- decideTests(ebFitLPS.ofB6LPSR3C8, method="global",
                          adjust.method="BH", p.value=0.05, lfc=0.59)

diffProbesLPS.ofB6LPSR3  <- which(resultsLPS.ofB6LPSR3[,1] !=0)
diffProbesLPS.ofB6LPSR3C8  <- which(resultsLPS.ofB6LPSR3C8[,1] !=0 | resultsLPS.ofB6LPSR3C8[,2] !=0)

library(Biobase)
myEset.ALL <- new("ExpressionSet", exprs = diffDataUT.LPS.B6)

# retrieve expression data for the probes from above
diffData.LPS.ofB6LPSR3  <- myEset.ALL[resultsLPS.ofB6LPSR3[,1] !=0]
diffData.LPS.ofB6LPSR3C8  <- myEset.ALL[resultsLPS.ofB6LPSR3C8[,1] !=0 |
                            resultsLPS.ofB6LPSR3C8[,2] !=0]

#pull the expression data back out of the eset object
diffData.LPS.ofB6LPSR3  <- exprs(diffData.LPS.ofB6LPSR3)
diffData.LPS.ofB6LPSR3C8  <- exprs(diffData.LPS.ofB6LPSR3C8)

#combine probeIDs, gene symbols and expression data for differentially expressed genes into one file
write.table(cbind(diffProbesLPS.ofB6LPSR3, diffData.LPS.ofB6LPSR3),
            "DiffGenesLPSofB6.B6vsR3.xls", sep="\t", quote=FALSE)
write.table(cbind(diffProbesLPS.ofB6LPSR3C8, diffData.LPS.ofB6LPSR3C8),
            "DiffGenesLPSofB6.vsR3C8.xls", sep="\t", quote=FALSE)
```

Generate heatmap of caspase-8-dependent genes regulated by LPS treatment.

```
#load gene set, consolidate replicates into averages and only show LPS-treated samples
head(diffData.LPS.ofB6LPSR3C8)
dim(diffData.LPS.ofB6LPSR3C8)
colnames(diffData.LPS.ofB6LPSR3C8) <- myGroups
diffData.LPS.ofB6LPSR3C8.AVG <- avearrays(diffData.LPS.ofB6LPSR3C8,
                            ID=colnames(diffData.LPS.ofB6LPSR3C8))
head(diffData.LPS.ofB6LPSR3C8.AVG)
dim(diffData.LPS.ofB6LPSR3C8.AVG)
diffData.LPS.subset.AVG <- diffData.LPS.ofB6LPSR3C8.AVG[,c(4,5,6)]
```

```
#draw heatmap of subset above
hr <- hclust(as.dist(1-cor(t(diffData.LPS.subset.AVG), method="pearson")), method="average")
hc <- hclust(as.dist(1-cor(diffData.LPS.subset.AVG, method="spearman")), method="complete")
dim(diffData.LPS.subset.AVG)
mycl <- cutree(hr, k=6)
mycolhc <- rainbow(length(unique(mycl)), start=0.1, end=0.9)
mycolhc <- mycolhc[as.vector(mycl)]
myheatcol <- greenred(75)
heatmap.2(diffData.LPS.subset.AVG, Rowv=as.dendrogram(hr), Colv=NA,
          col=myheatcol, scale="row", labRow=NA,
          density.info="none", trace="none", RowSideColors=mycolhc,
          cexRow=1, cexCol=1, margins=c(12,25), key=T)
```

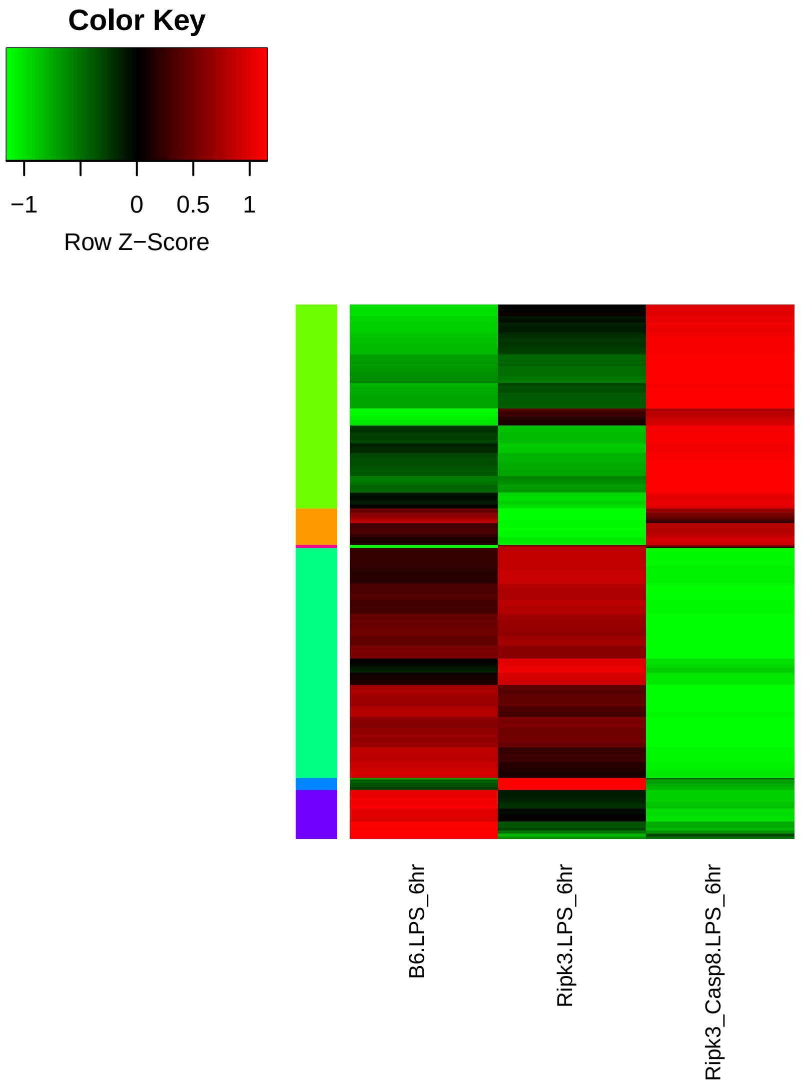


```
#now find out what your clusters are
names(mycolhc) <- names(mycl)
barplot(rep(10, max(mycl)),
        col=unique(mycolhc[hr$labels[hr$order]]),
```

```r
        horiz=T, names=unique(mycl[hr$order]), margins=c(12,25))
```

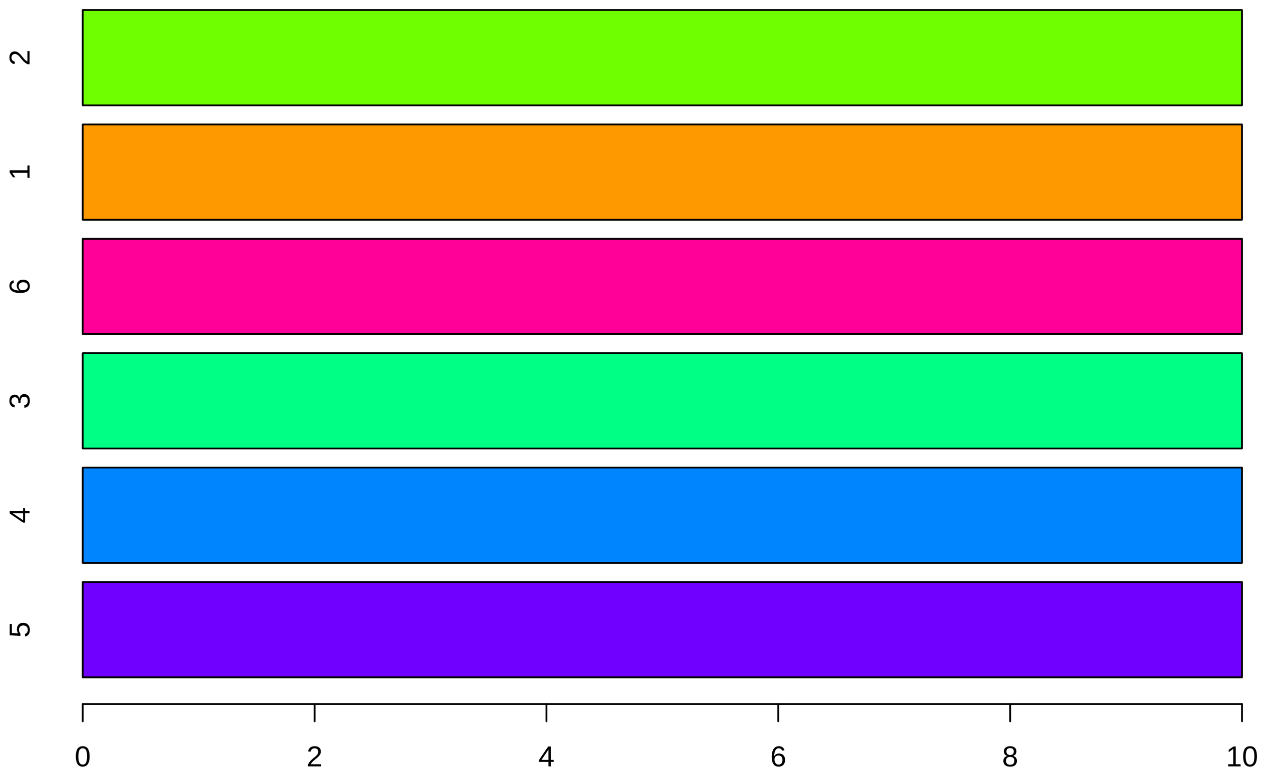


```r
#The numbers next to the color boxes correspond to the cluster numbers in 'mycl'.
#clusters 2,1,6,3,4,5 from top down for diffData.LPS.ofB6LPSR3C8.AVG

#select sub-clusters of co-regulated transcripts for downstream analysis c(2)=cluster 1
clid <- c(2)
ysub <- diffData.LPS.subset.AVG[names(mycl[mycl%in%clid]),]
hrsub <- hclust(as.dist(1-cor(t(ysub), method="pearson")), method="average")
clusterIDs <- data.frame(Labels=rev(hrsub$labels[hrsub$order]))
clusterIDs <- as.vector(t(clusterIDs))
heatmap.2(ysub, Rowv=as.dendrogram(hrsub), Colv=NA, labRow=NA, col=myheatcol,
          scale="row", density.info="none", trace="none",
          RowSideColors=mycolhc[mycl%in%clid], margins=c(12,25), cexCol=1)
```

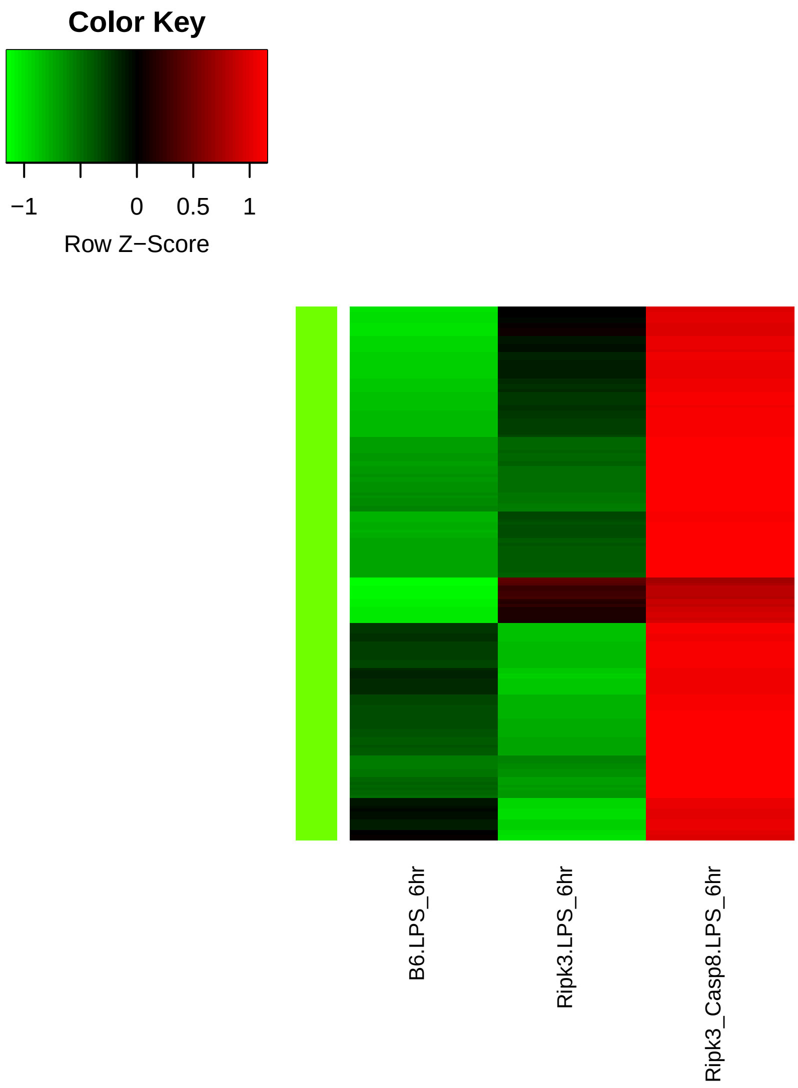


```r
#retrieve gene symbols for selected cluster for downstream applications (i.e. GO enrichment in DAVID)
write.table(clusterIDs, "LPSinducedR3C8.cluster1.xls", sep="\t", quote=FALSE)

#select sub-clusters of co-regulated transcripts for downstream analysis c(3)=cluster 2
clid <- c(3)
ysub <- diffData.LPS.subset.AVG[names(mycl[mycl%in%clid]),]
hrsub <- hclust(as.dist(1-cor(t(ysub), method="pearson")), method="average")
clusterIDs <- data.frame(Labels=rev(hrsub$labels[hrsub$order]))
clusterIDs <- as.vector(t(clusterIDs))
heatmap.2(ysub, Rowv=as.dendrogram(hrsub), Colv=NA, labRow=NA, col=myheatcol,
          scale="row", density.info="none", trace="none",
          RowSideColors=mycolhc[mycl%in%clid], margins=c(12,25), cexCol=1)
```

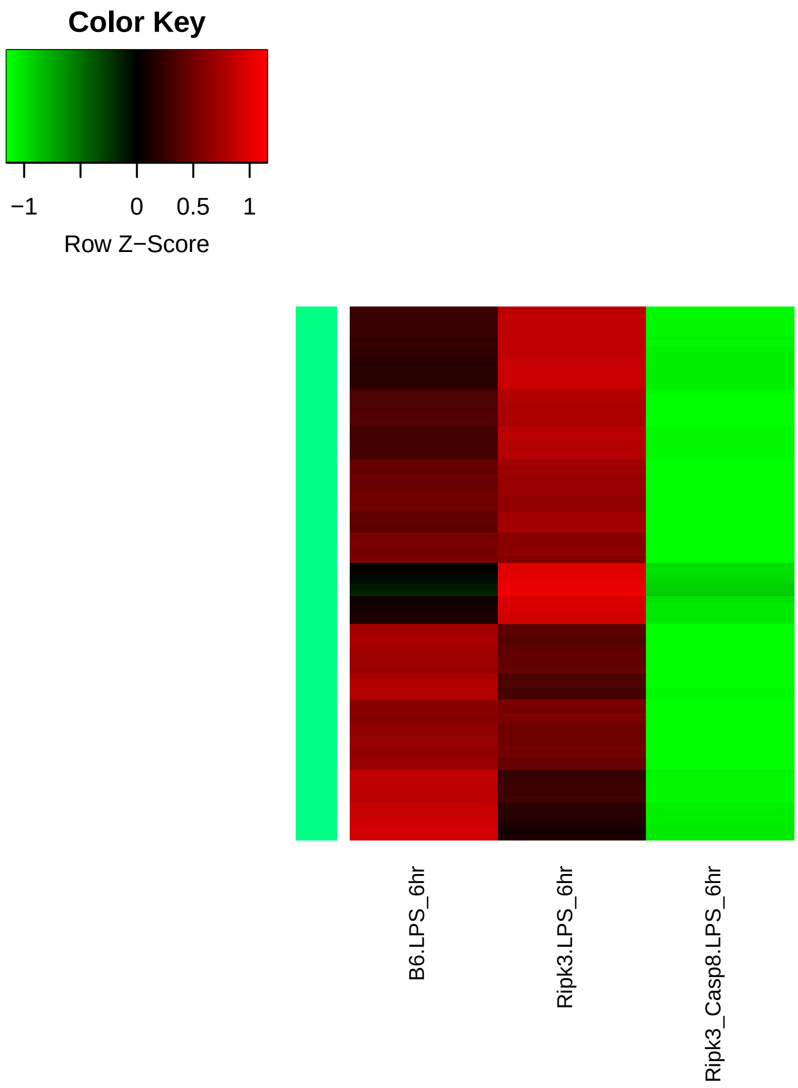


```
#retrieve gene symbols for selected cluster for downstream applications (i.e. GO enrichment in DAVID)
write.table(clusterIDs, "LPSinducedR3C8.cluster2.xls", sep="\t", quote=FALSE)
```

Generate heatmap of subset of cluster 2.

```
#read in your data from a text file that contains genes symbols as rows, and samples as columns.
#This file MUST be a dataframe, NOT a data matrix
myDiffGenes.LPS <- read.delim("LPS.Cluster2.txt", header=TRUE)
head(myDiffGenes.LPS)
colnames(myDiffGenes.LPS)
#use the dplyr 'mutate' command to get averages and fold changes for all your replicates
myDiffGenes.LPS <- mutate(myDiffGenes.LPS,
                  B6.untreated.AVG = (B6.untreated.1 + B6.untreated.2)/2,
                  Ripk3.untreated.AVG = (Ripk3.untreated.1 + Ripk3.untreated.2)/2,
                  Ripk3_Casp8.untreated.AVG = (Ripk3_Casp8.untreated.1 + Ripk3_Casp8.untreated.2)/2,
                  B6.LPS_6hr.AVG = (B6.LPS_6hr.1 + B6.LPS_6hr.2)/2,
                  Ripk3.LPS_6hr.AVG = (Ripk3.LPS_6hr.1 + Ripk3.LPS_6hr.2)/2,
                  Ripk3_Casp8.LPS_6hr.AVG = (Ripk3_Casp8.LPS_6hr.1 + Ripk3_Casp8.LPS_6hr.2)/2,
```

```
                   LogFC.B6.LPS_6hr.vs.B6.untreated = (B6.LPS_6hr.AVG - B6.untreated.AVG),
                   LogFC.Ripk3.LPS_6hr.vs.Ripk3.untreated = (Ripk3.LPS_6hr.AVG - Ripk3.untreated.AVG),
                   LogFC.Ripk3_Casp8.LPS_6hr.vs.Ripk3_Casp8.untreated =
                     (Ripk3_Casp8.LPS_6hr.AVG - Ripk3_Casp8.untreated.AVG),
                   LogFC.Ripk3.LPS_6hr.vs.B6.LPS_6hr = (Ripk3.LPS_6hr.AVG - B6.LPS_6hr.AVG),
                   LogFC.Ripk3_Casp8.LPS_6hr.vs.Ripk3.LPS_6hr =
                     (Ripk3_Casp8.LPS_6hr.AVG - Ripk3.LPS_6hr.AVG),
                   LogFC.Ripk3_Casp8.LPS_6hr.vs.B6.LPS_6hr = (Ripk3_Casp8.LPS_6hr.AVG - B6.LPS_6hr.AVG),
                   LogFC.Ripk3_Casp8.untreated.vs.Ripk3.untreated =
                     (Ripk3_Casp8.untreated.AVG - Ripk3.untreated.AVG),
                   LogFC.Ripk3.untreated.vs.B6.untreated = (Ripk3.untreated.AVG - B6.untreated.AVG),
                   LogFC.Ripk3_Casp8.untreated.vs.B6.untreated =
                     (Ripk3_Casp8.untreated.AVG - B6.untreated.AVG),
                   FC.Ripk3_Casp8.LPS_6hr.vs.Ripk3.LPS_6hr =
                     (-2^(-LogFC.Ripk3_Casp8.LPS_6hr.vs.Ripk3.LPS_6hr)),
                   FC.Ripk3_Casp8.LPS_6hr.vs.Ripk3_Casp8.untreated =
                     (2^(LogFC.Ripk3_Casp8.LPS_6hr.vs.Ripk3_Casp8.untreated)),
                   FC.Ripk3.LPS_6hr.vs.Ripk3.untreated = (2^(LogFC.Ripk3.LPS_6hr.vs.Ripk3.untreated)))

head(myDiffGenes.LPS)
#use dplyr "arrange" and "select" functions to sort by LogFC column of interest (arrange)
myDiffGenes.LPS <- myDiffGenes.LPS %>%
  arrange(desc(LogFC.Ripk3_Casp8.LPS_6hr.vs.Ripk3.LPS_6hr))
head(myDiffGenes.LPS)
write.table(myDiffGenes.LPS, "myGOCluster2.xls", sep="\t", quote=FALSE)

#Generate heatmap of Cluster2 subset
mySelectedHeatmap <- read.delim("LPS.Cluster2.heatmap.txt", sep="\t",
                    stringsAsFactors = FALSE, header=TRUE, row.names=1)
head(mySelectedHeatmap)
dim(mySelectedHeatmap)
colnames(mySelectedHeatmap) <- myGroups
mySelectedHeatmap.AVG <- avearrays(mySelectedHeatmap, ID=colnames(mySelectedHeatmap))
head(mySelectedHeatmap.AVG)
dim(mySelectedHeatmap.AVG)
mySelectedHeatmap.AVG <- mySelectedHeatmap.AVG[,c(4,5,6)]
mySelectedHeatmap <- as.matrix(mySelectedHeatmap.AVG)

hr <- hclust(as.dist(1-cor(t(mySelectedHeatmap), method="pearson")), method="average")
hc <- hclust(as.dist(1-cor(mySelectedHeatmap, method="spearman")), method="complete")
dim(mySelectedHeatmap)
mycl <- cutree(hr, k=1)
mycolhc <- rainbow(length(unique(mycl)), start=0.1, end=0.9)
mycolhc <- mycolhc[as.vector(mycl)]
myheatcol <- greenred(75)
#Rowv=NA maintains the order of the rows you have in your data table.
heatmap.2(mySelectedHeatmap, Rowv=NA, Colv=NA, col=myheatcol,
          scale="row", density.info="none", trace="none",
          RowSideColors=mycolhc, labRow=NULL,
          cexRow=1.5, cexCol=1, margins=c(12,25), key=T)
```

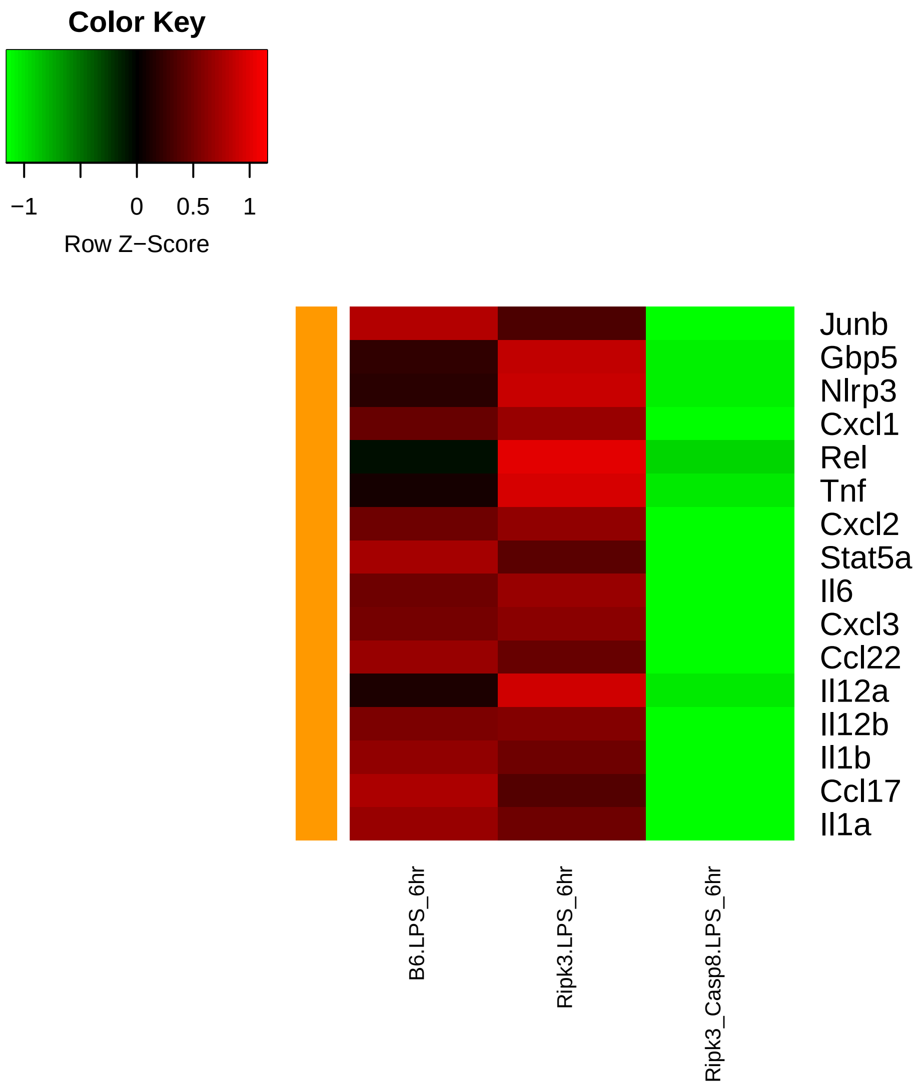

All analyses were carried out on the following system:

```
sessionInfo()
```

R version 3.2.2 (2015-08-14) Platform: x86_64-apple-darwin13.4.0 (64-bit) Running under: OS X 10.10.5 (Yosemite)

locale: [1] en_US.UTF-8/en_US.UTF-8/en_US.UTF-8/C/en_US.UTF-8/en_US.UTF-8

attached base packages: [1] stats4 parallel stats graphics grDevices utils datasets [8] methods base

other attached packages: [1] devtools_1.9.1 knitr_1.12
[3] rmarkdown_0.9.2 gplots_2.17.0
[5] RColorBrewer_1.1-2 ggvis_0.4.2
[7] dplyr_0.4.3 reshape2_1.4.1
[9] ggplot2_1.0.1 WGCNA_1.48
[11] fastcluster_1.1.16 dynamicTreeCut_1.62
[13] org.Mm.eg.db_3.2.3 RSQLite_1.0.0
[15] DBI_0.3.1 AnnotationDbi_1.32.0
[17] ShortRead_1.28.0 GenomicAlignments_1.6.1
[19] SummarizedExperiment_1.0.1 Biobase_2.30.0
[21] Rsamtools_1.22.0 GenomicRanges_1.22.1

[23] GenomeInfoDb_1.6.1 Biostrings_2.38.2
[25] XVector_0.10.0 IRanges_2.4.5
[27] S4Vectors_0.8.4 BiocParallel_1.4.0
[29] BiocGenerics_0.16.1 edgeR_3.12.0
[31] limma_3.26.3 Rsubread_1.20.2

loaded via a namespace (and not attached): [1] splines_3.2.2 foreach_1.4.3 gtools_3.5.0
[4] Formula_1.2-1 shiny_0.12.2 assertthat_0.1
[7] latticeExtra_0.6-26 yaml_2.1.13 impute_1.44.0
[10] lattice_0.20-33 digest_0.6.8 colorspace_1.2-6
[13] htmltools_0.2.6 httpuv_1.3.3 preprocessCore_1.32.0 [16] plyr_1.8.3 zlibbioc_1.16.0 xtable_1.8-0
[19] GO.db_3.2.2 scales_0.3.0 gdata_2.17.0
[22] lazyeval_0.1.10 nnet_7.3-11 proto_0.3-10
[25] survival_2.38-3 magrittr_1.5 mime_0.4
[28] memoise_0.2.1 evaluate_0.8 doParallel_1.0.10
[31] MASS_7.3-45 hwriter_1.3.2 foreign_0.8-66
[34] tools_3.2.2 formatR_1.2.1 matrixStats_0.15.0
[37] stringr_1.0.0 munsell_0.4.2 cluster_2.0.3
[40] lambda.r_1.1.7 caTools_1.17.1 futile.logger_1.4.1
[43] grid_3.2.2 iterators_1.0.8 labeling_0.3
[46] bitops_1.0-6 gtable_0.1.2 codetools_0.2-14
[49] R6_2.1.1 gridExtra_2.0.0 Hmisc_3.17-0
[52] futile.options_1.0.0 KernSmooth_2.23-15 stringi_1.0-1
[55] Rcpp_0.12.2 rpart_4.1-10 acepack_1.3-3.3
