# Supplementary material for: Activity of Uncleaved Caspase-8 Controls Anti-bacterial Immune Defense and TLR-Induced Cytokine Production Independent of Cell Death
Source: PLoS Pathog. 2016 Oct 13;12(10):e1005910. doi: 10.1371/journal.ppat.1005910 (PMC5063320; doi:10.1371/journal.ppat.1005910)
Supplement: S2 Table — Reference numbering refers to Supplemental References in S1 Text. (DOCX) [file ppat.1005910.s010.docx]

**Supplemental Table S2: Primer sequences for ChIP and RT-qPCR.**

| **Gene** | **Primer Name** | **Primer Sequence** | **Type** | **Reference** |
| --- | --- | --- | --- | --- |
| *Gapdh* | GapdF | GGTCCAAAGAGAGGGAGGAG | ChIP | ([3] |
| *Gapdh* | GapdR | GCCCTGCTTATCCAGTCCTA | ChIP | [3] |
| *Cxcl2* | Cxcl2F | GGGCTCTGTGCTTCCTGAT | ChIP | [3] |
| *Cxcl2* | Cxcl2R | TCCCGAGAGCTCCTTTTATG | ChIP | [3] |
| *Il1beta* | Il1betaF | CCCACCCTTCAGTTTTGTTG | ChIP | [3] |
| *Il1beta* | Il1betaR | CTTGTTTTCCCTCCCTTGTTT | ChIP | [3] |
| *Ccl5* | Ccl5F | CTGCTACCCTGGCTCCCTAT | ChIP | [3] |
| *Ccl5* | Ccl5R | TGGGAGATGCATGTGCTGT | ChIP | [3] |
| *Il6* | Il6F | AATGTGGGATTTTCCCATGA | ChIP | [3] |
| *Il6* | Il6FR | GCTCCAGAGCAGAATGAGCTA | ChIP | [3] |
| *Tnf* | TnfF | GATTCCTTGATGCCTGGGTGTC | ChIP | [3] |
| *Tnf* | TnfR | GAGCTTCTGCTGGCTGGCTGT | ChIP | [3] |
| *Il12b* | Il12bF | GGGGAGGGAGGAACTTCTTA | ChIP | [3] |
| *Il12b* | Il12bR | CTTTCTGATGGAAACCCAAAG | ChIP | [3] |
| *Hbbs* | HbbsF | GCATGGAAGACAGGACAATC | ChIP | This study |
| *Hbbs* | HbbsR | GTGGGAGGAGTGTACAAGGA | ChIP | This study |
| *Ifnb* | IfnbF | AGATGTCCTCAACTGCTCTC | Transcript | [4] |
| *Ifnb* | IfnbR | AGATTCACTACCAGTCCCAG | Transcript | [4] |
| *Il1b* | Il1bF | CCTCTGATGGGCAACCACTT | Transcript | [5] |
| *Il1b* | Il1bR | TTCATCCCCCACACGTTGAC | Transcript | [5] |
| *Il12b* | Il12bF | TTGAAAGGCTGGGTATCGGT | Transcript | [6] |
| *Il12b* | Il12bR | GAATTTCTGTGTGGCACTGG | Transcript | [6] |
| *Il6* | Il6F | ACAGAAGGAGTGGCTAAGGA | Transcript | [6] |
| *Il6* | Il6R | CGCACTAGGTTTGCCGAGTA | Transcript | [6] |
| *SeV NP* | SeV NPF | TGCCCTGGAAGATGAGTTAG | Transcript | [4] |
| *SeV NP* | SeV NPR | GCCTGTTGGTTTGTGGTAAG | Transcript | [4] |
| *Cxcl1* | Cxcl1F | GCACCCAAACCGAAGTCATA | Transcript | This study |
| *Cxcl1* | Cxcl1R | CTTGGGGACACCTTTTAGCA | Transcript | This study |
| *Cxcl2* | Cxcl2F | GATACTGAACAAAGGCAAGGC | Transcript | This study |
| *Cxcl2* | Cxcl2R | ATCAGGTACGATCCAGGCT | Transcript | This study |
| *Ccl22* | Ccl22F | GGCCATACAAAGTGATACCT | Transcript | This study |
| *Ccl22* | Ccl22R | GGAAGCAAGAATGGGTTCTA | Transcript | This study |
